# Resistance patterns, virulence determinants, and biofilm genes of multidrug-resistant *Pseudomonas aeruginosa* isolated from fish and fish handlers

**DOI:** 10.1038/s41598-024-73917-4

**Published:** 2024-10-14

**Authors:** Rasha M. M. Abou Elez, Eman Mohamed Fayek Zahra, Rasha M. A. Gharieb, Mohamed Elsayed Mohamed Mohamed, Mohamed Samir, Alaaeldin Mohamed Saad, Abdallah Mohamed Amin Merwad

**Affiliations:** Department of Zoonoses, Faculty of Veterinary Medicine, Zagazig University, Zagazig, Egypt

**Keywords:** Antibiotic resistance, Biofilm formation, Biofilm genes, *P. aeruginosa*, Virulence genes, Antimicrobials, Bacteria, Biofilms

## Abstract

*Pseudomonas aeruginosa* (*P. aeruginosa*) is an opportunistic bacterium that is widely distributed in aquatic environments and causes major economic losses in fish and public health hazards.This study aimed to identify the occurrence of *P. aeruginosa* in samples collected from fish and fish handlers, and to investigate the antimicrobial susceptibility, virulence determinants, and biofilm genes of *P. aeruginosa* isolates. A total of 276 samples were cross-sectionally collected from Nile tilapia (53), Golden grey mullet (52), Mediterranean horse mackerel (50), Striped red mullet (71), and fish handlers (50) at five different retail fish markets in Damietta Governorate, Egypt. *Pseudomonas* species (spp.) were biochemically identified in 57.9% of the total examined samples. *Peudomonas aeruginosa* were the most prevalent species isolated from the fish and human samples via PCR technique. *Peudomonas aeruginosa* isolates exhibited full resistance (100%) to tobramycin (TOB), gentamicin (CN), and colistin (CL), with a high level of susceptibility (88.5%) to imipenem (IPM) using the disk diffusion method. Most *P. aeruginosa* isolates (84.6%) exhibited drug resistance, with 61.5% were multidrug resistance (MDR) and 23.1% were extensive drug resistance (XDR). Most isolates had at least four virulence-associated genes (*las*B, *tox*A, *exo*U, and *opr*L) and three biofilm genes (*psI*A, *peI*A, and *las*R) by using uniplex PCR. The *las*I, and *rhl*R Quorum Sensing (QS) genes were identified in 84.6% and 61.5% in the examined *P. aeruginosa* isolates, respectively. The highest mortality rate in Nile tilapia experimentally infected with *P. aeruginosa* isolate encoding most of virulent genes. Multivariate analyses revealed high heterogeneity among the examined isolates. This study revealed the emergence of virulent and drug resistant *P. aeruginosa* isolates in fish, poses high risks to consumers and food. Thus, strict hygienic measures should be considered when catching, handling, and storing fish, in addition to the routine application of antimicrobial susceptibility testing.

**Supplementary Information:**

The online version contains supplementary material available at 10.1038/s41598-024-73917-4.

## Introduction

Fish are a source of high-quality protein and metabolic nutrients such as omega-3 fatty acids, iodine, selenium, and vitamin D ^1,2^. Similarly, compared with that of meat products, fish consumption has recently increased by 3.2% per year, especially in developing countries^3^. Fish are more susceptible to bacterial deterioration than other meats due to a decreased pH and moisture content^4^. *Pseudomonas* spp. are the most frequently isolated bacteria from spoiled fish that lead to low-quality fish products because they are part of the normal fish microbiota^4^. The *Pseudomonas* genus contains more than 200 species, including *Pseudomonas aeruginosa* (*P. aeruginosa*), *Pseudomonas fluorescens* (*P. fluorescens*), *Pseudomonas lundensis* (*P. lundensis*), *Pseudomonas fragi* (*P. fragi*), *Pseudomonas anguilliseptica* (*P. anguilliseptica*), and *Pseudomonas putida* (*P. putida*)^5^. These bacteria are widespread in aquatic environments, both in freshwater and marine water^3^. Fish transport microorganisms from their natural aquatic environment and contaminate utensils while handling and contacting fish handlers or workers with infected skin lesions^6,7^. *Pseudomonas* infection can be transmitted to humans, particularly immunocompromised individuals, through contact or consumption of contaminated raw fish^8^. *Pseudomonas aeruginosa* is an opportunistic gram-negative food spoilage bacterium that leads to nosocomial infections, particularly respiratory tract infections, gastrointestinal infections, systemic infections, and wound infections, in immunosuppressed individuals^9^. These bacteria can change from a commensal to a virulent form, leading to subsequent chronic disease in individuals at risk^9^, and harboring multidrug resistance features that can be transported to other human and animal pathogens^10^.

The prevalence of multidrug resistance (MDR) bacteria, which are considered a public health threat, has been increased all over the world. Several recent investigations have reported the emergence of MDR and extensive drug resistance (XRD) from different origins, increasing the necessity of the proper use of antibiotics. In addition, antimicrobial susceptibility testing as well as the screening of the emerging MDR strains, is routinely applied to detect the antibiotic of choice^11,12^. These MDR bacteria can resist antimicrobial drugs through mutations in chromosomal genes or by horizontal acquisition of resistance genes^13^. In addition, this bacterium is MDR and has diverse virulence determinants; biofilms enhance the colonization of *P. aeruginosa* by the host, reduce the efficacy of antimicrobial treatments, and subsequently increase the severity of *Pseudomonas* infection^13^.Biofilm producing *P. aeruginosa* is the key for its chronic colonization in human and animal tissues^14^. Biofilms assist exchange of antibiotic resistance genes between bacteria by supporting a physical barrier to antimicrobial agents and host immune response^15^.

The pathogenicity of *P. aeruginosa* is primarily linked to a variety of virulence genes, such as lipopolysaccharide (LPS), exopolysaccharides (alginate, *psl* and *pel*), cytotoxins (*exo*U, *exo*T, *exo*S and *exo*Y),, and toxin A (*tox*A), as well as elastase genes A and B (*las*A and *las*B), alkaline protease (*apr*A) LipC lipases, phospholipase C, and esterase A^16^. LPS can play a role in antibiotic tolerance and biofilm formation *psl* and *pel*, 20. Cytotoxins (*exo*U, *exo*T, *exo*S, and *exo*Y) can block phagocytosis and bacterial clearance. Exotoxin A (ETA) can reduce host protein synthesis through ADP ribosylation^17^. These genes increase intracellular oxidative effects and play a role in the adhesion, attachment, and invasion of host cells^18^. *Peudomonas aeruginosa* produces three essential exopolysaccharides implicated in biofilm formation, such as alginate, the polysaccharide synthesis locus (PSL), the pellicle (PEL) polysaccharide, and Quorum Sensing (QS), which play a role in the formation of mutants deficient in the pellicle, maintaining interactions between cell surfaces in biofilms and controlling the production of *las* and *rhl* virulence genes^19^. Molecular techniques are essential for the rapid detection of *P. aeruginosa* by amplification of species-specific primers, especially 16 S r*DNA* gene sequencing^20^.

The purposes of this study were to (i) determine the prevalence of *Pseudomonas* spp. in Nile tilapia, Golden grey mullet, Mediterranean horse mackerel, Striped red mullet and fish handlers at different retail fish markets in Damietta Governorate, Egypt; (ii) identify the antimicrobial resistance profiles, serotypes, virulence genes, biofilm genes, and QS genes of the recovered *P. aeruginosa* isolates, (iii) illustrate the genetic relatedness of XDR isolate using *16 S rDNA* gene sequencing, (iiii) assess the pathogenicity of virulent *P. aeruginosa* isolates on Nile tilapia with monitoring of morbidity and mortality rates, and (iiiii) investigate the diversity and correlation among *P. aeruginosa* isolates using multivariate statistical analyses.

## Materials and methods

### Study design and sampling

**Fig. 1:**
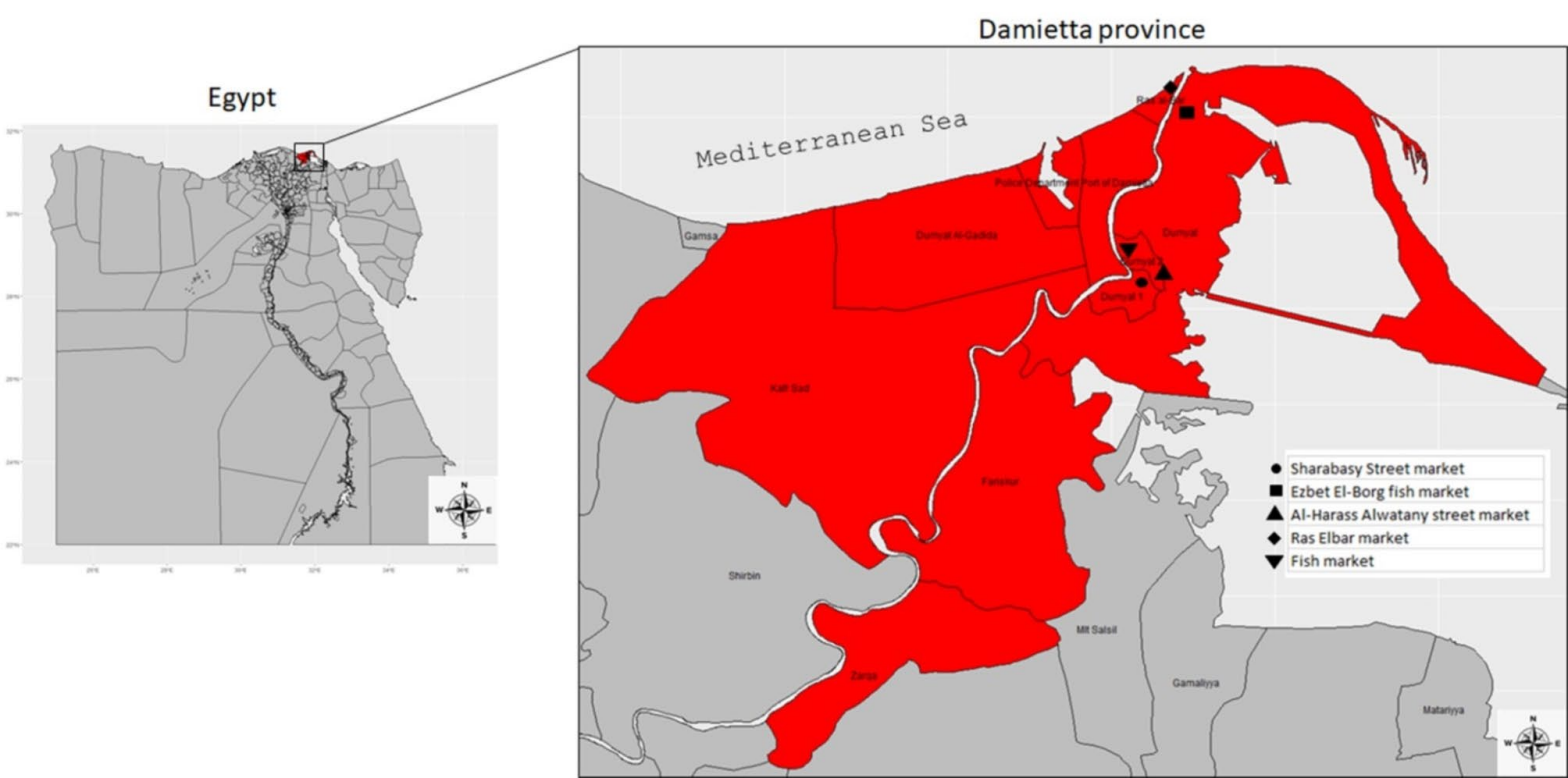
Map showing the sampling locations (fish markets) inside Damietta province, Egypt.

A cross-sectional study collected 276 apparently healthy fish samples with no clinical signs of infection from 105 freshwater fish (53 Nile tilapia/*Oreochromis niloticus* and 52 grey mullet/*Liza auratus*), 121 marine water fish (50 Mediterranean mackerel/*Trachurus mediterraneus* and 71 Striped red mullet/*Mullus surmuletus*), and 50 fish handlers at five different fish markets in Damietta governorate, Egypt as shown in Fig. 1, between January 2021 and May 2022. Sterile cotton swabs were used to collect samples from the fish handlers. The swabs were gently rubbed through the human seller’s hand before being immersed in tubes containing 9 mL of sterile buffered peptone water (BPW; Oxoid Ltd.,Basingstoke, Hampshire, UK). The samples were labeled with the market ID, sample type, and date of immediate transport to the laboratory for further processing and bacteriological analysis. Fish samples (muscle and liver) were processed and prepared following the procedures standardized by the International Organization for Standardization^21^. Clinical examination of fish for the presence of external and internal lesions was carried out as described previously^22^. The study design and sampling procedures were approved by the Institutional Animal Care and Use Committee (IACUC) of Zagazig University, Egypt (Ref. No.:ZU-IACUC/3/F/14/2024). All procedures involving animals were performed in accordance with the ARRIVE guidelines (PLoS Bio 8(6), e1000412, 2010). However, procedures involving human participants were performed in accordance with the 1964 Declaration of Helsinki and its later amendments or comparable ethical standards. In addition, written informed consent was obtained from the fish handlers who participated in the study.

### Isolation and identification of *Pseudomonas* spp.

The isolation procedures were performed according to ISO 3720^23^. The prepared fish samples (liver, muscle) were processed. For every sample, the amount of one gram of sample was added into 9 mL of 1% (w/v) BPW. The mixture was then homogenized for 30 s using a homogenizer and fish samples (1 mL) and human hand swabs were aseptically inserted in 9 mL sterile BPW followed by incubation at 25 °C for 44 h ± 4 h. A loopful from the incubated broth was then streaked on *Pseudomonas* agar base supplemented with *Pseudomonas* cephalothin-sodium fusidate-cetrimide (CFC) agar (Oxoid Ltd.,Basingstoke, Hampshire, UK), and incubated at 25 °C for 44 h ± 4 h. The presence of blue‒green/brown pigmentation or fluorescence indicates presumptive evidence of *Pseudomonas* spp. Colony morphology was identified by Gram staining. Species identification was determined by standard biochemical tests; catalase, oxidase, urease, indole, methyl red, Voges Proskauer, citrate utilization, H2S production, mannitol fermentation, Arginine hydrolysis, gelatin hydrolysis, nitrate reduction test sugar fermentation test, proteolytic activity, lipolytic activity, and motility test^24^. *Pseudomonas aeruginosa* isolates were serologically identified by the slide agglutination technique using 4 commercially available polyvalent and 16 monovalent antisera according to the manufacturer’s recommendation (Bio-Rad^®^, France) according to Glupczynski et al.^25^, and serotypes were determined based on the International Antigen Typing Scheme (IATS) according to Legakis et al.^26^. All *P. aeruginosa* isolates were subsequently confirmed via PCR via 16 S r*DNA* gene amplification^27^. DNA was extracted using a QIAamp DNA Mini Kit (Qiagen, Hilden, Germany) following the manufacturer’s instructions. The primer sequences (Metabion, Germany) and PCR cycling conditions are provided in Table S 1.

### Antimicrobial susceptibility test of *P. aeruginosa* isolates

All molecularly identified *P. aeruginosa* isolates (*n* = 26) were tested for susceptibility to 15 antibiotics using the Kirby–Bauer disk diffusion method following the Clinical and Laboratory Standards Institute guidelines^28^. Fifteen antibiotic disks (Thermo Fisher Scientific, Oxoid Ltd., UK) included tobramycin (TOB, 10 µg), gentamicin (CN, 10 µg), amikacin (AK, 30 µg), meropenem (MEM, 10 µg), doripenem (DOR, 10 µg), imipenem (IPM, 10 µg), ceftazidime (CAZ, 30 µg), cefepime (FEP, 30 µg), levofloxacin (LEV, 5 µg), ciprofloxacin (CIP, 5 µg), colistin (CT, 25 µg), piperacillin (PRL, 30 µg), Ampicillin (AMP, 10 µg), aztreonam (ATM, 30 µg) and amoxicillin – clavulanic acid (AMC, 30 µg). Briefly, the bacterial suspension was prepared in sterile saline and adjusted to the turbidity of the 0.5 McFarland standard. The suspension was then streaked on Muller–Hinton agar plates (Thermo Fisher Scientific, Oxoid Ltd., UK) using sterile swabs. Antibiotic discs were inserted on the surface of the plates, which were subsequently incubated at 35 °C ± 2 °C for 16–18 h. *P. aeruginosa* ATCC^®^ 27853 was used as a quality control organism. The inhibition zone diameter was measured, and the susceptibility or resistance of the organism to each drug was determined based on CLSI guidelines^28^. Multiple antibiotic resistance (MAR) was determined using the formula: a/b (where “a” is the number of antimicrobial agents to which an isolate was resistant and “b” is the total number of antimicrobial agents tested) following the protocol proposed by Krumperman^29^.Interpretation of antibiotic susceptibility to evaluate MDR, XDR and pan drug resistance (PDR) isolates was grouped according to Magiorakos et al.^30^.

### Phenotypic screening of biofilm-producing *P. Aeruginosa*

All *P. aeruginosa* isolates (*n* = 26) were tested for their ability to form biofilms using the microtiter plate assay^31^. In this assay, A bacterial suspension was prepared in Mueller Hinton broth supplemented with 1% glucose and adjusted to 0.5 McFarland (1.5 × 10^8^ CFU/mL). A 100 µL of the bacterial suspension was inoculated into sterile polystyrene microtiter plates. The plate was incubated at 37 °C for 24 h. Three wells containing only broth were left as negative controls in the plate. To remove free-floating cells, the media from the plate wells were discarded and washed twice with 0.2 mL of phosphate-buffered saline (PBS, pH 7.2). The plate was inverted, and PBS was removed by blotting with paper towels. The biofilm was fixed by adding 150µL of ethanol for 20 min, and the cells adhered to the microtiter plate were stained with 150µL of crystal violet for 15 min at room temperature after removing the stain. The wells were washed twice with PBS and air dried for 1 h. Biofilm quantification was carried out by adding 150µL of 95% ethanol to each well for 45 min. The optical density (OD) was then measured at a wavelength of 570 nm (OD_570_) using an ELISA reader (Sunrise, Tecan) after adjusting to the negative control (OD_c_) at zero. Mean and standard deviation of OD values were recorded for all *P. aeruginosa* isolates and negative controls. The cut of values were calculated by using the following formula:

OD_c_ = mean OD_c_ + (3 × standard deviation (SD) of negative control).

OD isolate = mean OD of isolate − OD_c_.

The isolates were then classified as negative (OD_570_ ≤ OD_c_), weak (OD_c_ < OD_570_ ≤ 2 × OD_c_), moderate (2 × OD_c_ < OD_570_ ≤ 4 × OD_c_), or strong (4 × OD_c_ < OD_570_) biofilm formers^32^. *P. aeruginosa* ATCC^®^ 27853 was used as the standard strain. The experiment was performed in triplicate.

### Molecular identification of virulence-, biofilm-associated genes and QS genes in *P. aeruginosa*

All *P. aeruginosa* isolates were amplified from the 16 S r*DNA* gene (*n* = 26) and were screened for the presence of virulence genes (*las*B, *tox*A, *exo*U, *opr*L) using uniplex PCR^20,33–35^. In addition, uniplex PCR was also conducted to detect the presence of biofilm-associated genes (*psl*A, *pel*A, *las*R)^36,37^ and QS genes (*las*I, *rhl*R)^37,38^ in all the biofilm-producing *P. aeruginosa* isolates (*n* = 26). The primer sequences (Metabion, Germany) for all the tested genes are provided in Table S 1. The PCR mixture (25 µl) for each tested gene consisted of 2x premix Emerald Amp GT PCR master mix (12.5 µl) (Takara Bio Inc., Shiga, Japan), PCR grade water (4.5 µl), forward and reverse primers (µl, each) and template DNA (1 µl). DNA amplification was performed in a T3 thermal cycler (Biometra), and the PCR cycling conditions are provided in Table S 1. The amplified products, positive (*P. aeruginosa* ATCC^®^ 27853) and negative controls for each target gene, along with a DNA ladder (Fermentas), were loaded onto an ethidium bromide (0.5 µg/ml)-stained agarose gel (1.5%) and run for 30 min. The resulting gel was photographed by a gel documentation system (Alpha Innotech), and the data were analyzed through computer software.

### DNA sequencing and phylogenetic analysis

The DNA products amplified from five XDR and strong biofilm-forming *P. aeruginosa* isolates with keys of H22, R18, K9, M6 and N1 as provided in Table 2 were isolated with a QIAquick PCR Kit (QIAGEN, Valencia, CA, USA, cat-number 4336817), following the manufacturer’s guidelines. *16 S rDNA* gene sequencing was carried out with primers (Table S 1) and an automated sequencer, as previously described^27^. The obtained sequences were resolved with the Basic Local Alignment Search Tool (BLAST^®^ analysis) of Informative Biotechnology website on the National Center. Gene sequences of the isolates were submitted to the GenBank sequence database under accession numbers PQ252928, PQ252932, PQ256806, PQ256807, and PQ256808. Initial BLAST^®^ analysis was performed to establish sequence identity with GenBank accessions^39^. These sequences were then aligned with others available in the GenBank sequence database. A comparative analysis of sequences was performed using the CLUSTAL W multiple sequence alignment program, version 12.1 of MegAlign module of Lasergene DNAStar software Pairwise (Madison, Wisconsin, USA) which was designed by Thompson et al.^40^. A phylogenetic tree was constructed using MEGA 6 ^41^.

### Pathogenicity testing

The pathogenicity of virulent *P. aeruginosa* isolates was assessed by the challenge of *O. niloticus*. A total of 120 apparently healthy *O. niloticus* weighting 45 ± 3 g with no history of previous infections were collected randomly from Fish Research Unit, Zagazig University, Egypt, and left acclimated in a clean twelve 60 L glass tank (10 fish / tank) with dechlorinated water flow, continuous aeration for 14 days prior to the challenge. *Oreochromis niloticus* was selected as a model for the present study due to its local availability and ease of cultivation, handling, and transportation. The tank was filled with sand-filtered, UV-sterilized, dechlorinated tap water with an average salinity of 0.3 ± 0.1 g L^ −1^. Dissolved oxygen was monitored at 5 ± 1 mg L^ −1^ using automatic air suppliers (RINA, Genova, Italy), while the water temperature was maintained at 27 ± 0.52 °C. Tank pH was regulated at 7.5 and 13 h light/11 h dark cycle was adopted. Water temperature, dissolved oxygen, and PH were monitored daily. Ammonia and nitrite were measured twice a week and never exceeded 0.05 and 0.25 mg L^ −1^ respectively. The fish were fed two times daily (09:00 and 20:00 h) until visual satiety on a commercial pellet of 30% crude protein (Skretting, Alexandria, Egypt). The organic wastes and other debris were siphoned and 30% of the water was replaced daily to reduce the toxicity of ammonia. The experiment was divided into four tank groups (30 fish per group) labelled G1, G2, G3, and G4. In group G1, the fish were inoculated intraperitoneally (IP) with 0.1 ml of sterile phosphate-buffered saline (PBS) and served as a control, while the fish of the other 3 groups (G2, G3 and G4) were injected IP with 0.1 ml of the overnight culture of virulent *P. aeruginosa* strains (M6, N1 and K10, respectively) at a concentration of 3 × 10^7^CFU/ml^42^. The isolates were selected based on harboring variable virulence genes. One of these isolates (M6) harbored *opr*L, *exo*U, *tox*A, and *las*B virulent genes, the other isolate (N1) encoded the *opr*L, *exo*U, and *las*B genes, while the (K10) isolate encoded *exo*U, *tox*A, and *las*B. For preparation of inoculum, the isolates were individually cultured in tryptic soy broth for 24 h at 37 °C. The growing bacteria were adjusted to 0.5 McFarland standards before IP injection. The clinical signs, cumulative mortalities, and postmortem lesions were recorded for each experimental groups daily for 2 weeks post-challenge. Mortalities were only considered by re-isolation of the injected isolates from the moribund and freshly dead fish. To compare the survivability of fish belonging to various virulence groups, Kaplan-Meier analysis (including the plot) was done. The overall and pairwise differences among the groups were determined for significance using both Log-rank (Mantel-Cox) and Log-rank for trend tests. This analysis was done using GraphPad Prism software v. 8. The fish experiment was carried out following guidelines and regulations for animal experiments, and was approved by the Institutional Committee of Animal Care and Use at Zagazig University (approval number: ZU-IACUC/3/F/14/2024).

### Multivariate statistics and machine learning

To visualize the overall distribution of AMR phenotypes, virulence, biofilm genes, and bacterial serotypes in the isolates and thus estimate the clustering among isolates, we generated a heatmap supported by a dendrogram using their binary data. This was performed using the R package heatmap^43^. To determine and visualize the dissimilarities of the associations among isolates from different hosts/organs, we performed a nonmetric multidimensional scaling analysis based on Bray‒Curtis (Sorensen) distances, using the R package vegan and the function metaMDS^44^. The significant differences among groups were estimated using the PERMANOVA test^45^. A random forest (RF) classification model was used to rank bacterial features that are discriminatory for isolates from various hosts (i.e., humans, freshwater fish, and marine fish) using the mean decrease in accuracy. In this model, prediction was based on the majority of votes in an ensemble of 500 trials and a bag error of 0.6. Fisher’s exact test was used to determine whether there were significant differences in the frequency of bacterial features between humans, freshwater fish, and marine fish. This analysis was performed using the metaboanalyst platform^46^ R software was used to detect correlations among various features using the R package Hmisc^47^.

## Results

### Phenotypic characteristics and occurrence of *Pseudomonas* spp. in fish and fish handlers

A total of 276 apparently healthy fish samples were examined for the presence of external and internal lesions. No signs of external or internal lesions were observed in all examined fish samples. All isolated *Pseudomonas* spp. were blue-green or brown pigmentation or fluorescence colonies on *Pseudomonas* agar base supplemented with *Pseudomonas* CFC agar on bacteriological examination. Biochemically, identified *P. aeruginosa* isolates (*n* = 160) were positive for oxidase, mannitol fermentation, gelatin hydrolysis, catalase, citrate utilization, and nitrate reduction test, but were negative for methyl red, H2 S production, indole, urease, and Voges-Proskauer tests. *Pseudomonas* spp. were biochemically identified in 160 (57.9%) of the total examined samples, constituting 32 (60.4%) from Nile tilapia, 34 (65.4%) from Golden grey mullet, 29 (58%) from Mediterranean horse mackerel, 42 (59.2%) from Striped red mullet and 23 (46%) from fish handlers (Table S 2 and Table 1). All isolated *Pseudomonas* spp. were more frequently isolated from the liver samples of the total examined fish samples (37.6%) than from the muscle samples (23%), as presented in Table S 2. Twelve *Pseudomonas* spp. were identified as *P. aeruginosa*,* P. fluorescens*,* P. alcaligenes*,* P. stutzeri*,* P. fragi*,* P. brenneri*,* P. psychrophila*,* P. oryzihabitans*,* P. putida*,* P. lundensis*,* P. proteolytica* and *P. luteola* in this study using standard biochemical tests (Table 1). *P. aeruginosa* and *P. fluorescens* were the prevalent isolated species in the fish and human samples, as detailed in Table 1.

**Table 1:** Occurrence of *Pseudomonas* spp. (*n* = 160) in fish and fish handlers.

Sample source	No. positive samples (%)	*P. aeruginosa*	*P. fluorescens*	*P. alcaligenes*	*P. stutzeri*	*P. fragi*	*P. brenneri*	*P. psychrophila*	*P. oryzihabitans*	*P. putida*	*P. lundensis*	*P. proteolytica*	*P. luteola*
Nile tilapia^F^ (*n* = 53)	32 (60.3)	5 (15.6)	13 (40.6)	4 (12.5)	2 (6.3)	3 (9.8)	1 (3.1)	0 (0.0)	1 (3.1)	1 (3.1)	1 (3.1)	1 (3.1)	0 (0.0)
Golden grey mullet^F^ (*n* = 52)	34 (65.3)	3 (8.8)	9 (26.5)	2 (5.9)	0 (0.0)	7 (20.6)	0 (0.0)	4 (11.8)	1 (2.9)	2 (5.9)	0 (0.0)	3 (8.9)	3 (8.8)
Mediterranean mackerel^M^ (*n* = 50)	29 (58.0)	5 (17.2)	8 (27.6)	2 (6.9)	2 (6.9)	5 (17.2)	1 (3.5)	3 (10.3)	0 (0.0)	1 (3.5)	1 (3.5)	1 (3.5)	0 (0.0)
Striped red mullet^M^ (*n* = 71)	42 (59.2)	8 (19.1)	18 (42.9)	3 (7.1)	4(9.5)	5 (11.9)	1 (2.4)	0 (0.0)	0 (0.0)	2 (4.8)	1 (2.4)	0 (0.0)	0 (0.0)
Human (*n* = 50)	23 (46.0)	5 (21.7)	7 (30.4)	2 (8.7)	0 (0.0)	1 (4.4)	1 (4.4)	0 (0.0)	0 (0.0)	3 (13.0)	0 (0.0)	2 (8.7)	2 (8.7)
Total	160 (57.9)	26 (16.3)	55 (34.4)	13 (8.1)	8 (5.0)	21 (13.1)	4 (2.5)	7 (4.4)	2 (1.3)	9 (5.6)	3 (1.9)	7 (4.4)	5 ( 3.1)

### Serotyping of *P. aeruginosa* isolates

Seven different serotypes of the recovered *P. aeruginosa* were identified, representing six serogroups, as presented in Table 2. Serotype O11, which belongs to group E, was the most common isolated serotype (30.7%) (Table 2). However, serotype O10 belonged to Group H, serotype O2 belonged to Group B (19.2% each), serotype O4 belonged to Group F (11.5%), serotype O5 belonged to Group B, serotype O8 belonged to Group C (7.7% each), and serotype O6 belonged to Group G (3.8%). These were the remaining isolated serotypes (Table 2).

**Table 2:** Serotypes, virulotypes, antibiotic resistance profiles, biofilm genes and biofilm degree of *P. aeruginosa* isolates (*n* = 26).

ID	Source^1^	Serotype^2^	Virulence genes^3^				Antibiotic resistance^4^		Biofilm genes			Biofilm grade^5^	QS genes	
			LasB	ToxA	ExoU	OprL	Phenotypic resistance and patterns	MAR index	PslA	PelA	LasR		lasI	rhlR
N1	Tilapia ^M^	011^E^	+	−	+	+	CT, TOB, CN, AK, PRL, CAZ, LEV, FEP, CIP, MEM, DOR, ATM, AMC, ^X^/ 8classes	0.867	−	+	+	S	+	+
N2	Tilapia ^M^	O10^H^	+	−	+	+	CT, TOB, CN, AK, CAZ, AMP, AMC^M^/ 4 classes	0.467	+	+	+	S	+	+
N3	Tilapia ^L^	O11^E^	+	−	+	+	CT, TOB, CN, AK, CAZ, LEV, FEP, CIP^M^/ 4 classes	0.533	+	+	+	S	+	+
N4	Tilapia ^L^	O10^H^	+	−	+	+	CT, TOB, CN, AK, CAZ, LEV, PRL, FEP^M^/ 6 classes	0.533	+	+	+	S	+	+
N5	Tilapia ^L^	O5^B^	+	−	+	+	CT, TOB, CN, AK, CAZ, AMP, AMC^M^ /4 classes	0.467	+	+	+	S	+	+
M6	G Mullet ^L^	O2^B^	+	+	+	+	CT, TOB, CN, AK, CAZ, LEV, PRL, FEP, CIP, MEM, DOR, ATM, AMP, AMC^X^/ 8 classes	0.933	+	+	+	S	+	+
M7	G Mullet ^M^	O4^F^	+	+	+	−	CT, TOB, CN, AK, CAZ^M^/ 3 classes	0.333	+	+	+	S	+	+
M8	G Mullet ^M^	O4^F^	+	+	+	+	CT, TOB, CN	0.200	+	−	−	W	+	−
K9	Mackerel ^M^	O11^E^	+	+	+	+	CT, TOB, CN, AK, CAZ, LEV, PRL, FEP, CIP, AMP, AMC ^X^/ 6 classes	0.733	+	+	+	S	+	+
K10	Mackerel ^M^	O10^H^	+	+	+	−	CT, TOB, CN, AK	0.267	+	+	−	M	−	−
K11	Mackerel ^L^	O11 ^E^	+	−	+	+	CT, TOB, CN, AK, CAZ, LEV, PRL, AMP, AMC^M^/ 6 classes	0.600	+	+	+	S	+	+
K12	Mackerel ^L^	O6^G^	+	+	+	+	CT, TOB, CN, AMP, AMC^M^/ 4 classes	0.333	+	−	−	W	+	−
K13	Mackerel ^L^	O10 ^H^	+	+	+	+	CT, TOB, CN, AK, CAZ^M^/ 3 classes	0.333	+	+	+	S	+	−
R14	R mullet ^M^	O8 ^C^	+	+	+	+	CT, TOB, CN, AK, CAZ, LEV, PRL, FEP, CIP, MEM, DOR, ATM, IPM ^X^/ 7 classes	0.867	+	+	+	S	+	−
R15	R mullet ^M^	O11 ^E^	+	+	+	+	CT, TOB, CN, AK, CAZ, LEV, PRL, AMP, AMC ^M^/ 6 classes	0.600	+	+	+	S	+	+
R16	R mullet ^M^	O8 ^C^	+	+	+	+	CT, TOB, CN, AK	0.267	+	+	−	M	−	−
R17	R mullet ^M^	O10 ^H^	+	+	+	+	CT, TOB, CN, AK, CAZ^M^/ 3 classes	0.333	+	+	+	S	+	−
R18	R mullet ^L^	O2 ^B^	+	+	+	+	CT, TOB, CN, AK, CAZ, LEV, PRL, FEP, CIP, MEM, DOR, ATM, AMP, AMC ^X^/ 8 classes	0.933	+	+	+	S	+	+
R19	R mullet ^L^	O2 ^B^	+	+	+	+	CT, TOB, CN, AK	0.670	+	−	+	M	−	−
R20	R mullet ^L^	O11 ^E^	+	+	+	−	CT, TOB, CN, AMP, AMC^M^/ 4 classes	0.333	+	−	−	W	+	−
R21	R mullet ^L^	O4 ^F^	+	+	+	+	CT, TOB, CN, AK, CAZ^M^/ 3 classes	0.333	+	+	+	S	+	+
H22	Human ^S^	O2 ^B^	+	+	+	+	CT, TOB, CN, AK, CAZ, LEV, PRL, FEP, CIP, MEM, DOR, ATM, AMC^X^8 classes	0.867	+	+	+	S	+	+
H23	Human ^S^	O11 ^E^	+	+	+	+	CT, TOB, CN, AK, CAZ^M^/ 3 classes	0.333	+	+	+	S	+	+
H24	Human ^S^	O2 ^B^	+	+	+	+	CT, TOB, CN, AK, CAZ, LEV, PRL, FEP, CIP, MEM, DOR, AMC ^M/^/7 classes	0.800	+	+	+	S	+	+
H25	Human ^S^	O11 ^E^	+	+	+	+	CT, TOB, CN, AK, CAZ, AMC^M^/ 4 classes	0.400	+	+	+	S	+	+
H26	Human ^S^	O5 ^B^	+	+	+	+	CT, TOB, CN, AMP, AMC^M^/ 4 classes	0.333	+	−	−	M	−	−

^1^M: G mullet: Golden grey mullet (*Mugil auratus*), R mullet: Striped red mullet (*Mullus surmuletus*), M: muscle, L: liver, S: hand swab, ^2^serogroup, ^3^ +: virulence genes positive, −: virulence genes negative, ^4^M: multiple drug resistant, X: extensive drug resistant, MAR: multiple antibiotic index, ^5^W: weak biofilm producer, M: moderate biofilm producer, S: strong biofilm producer.

### Antimicrobial susceptibility of *P. aeruginosa* isolates

The patterns of antibiotic resistance of the *P. aeruginosa* isolates to the 15 antibiotics are presented in Tables 2 and 3. *P. aeruginosa* isolates exhibited full resistance to TOB, CN, and CL (100%). Notably, the isolates demonstrated an elevated degree of resistance to AK (84.6%) and CAZ (73.1%). Reciprocally, the isolates displayed a high level of susceptibility to IPM (88.5%) and ATM (80.8%). Most isolates (84.6%) exhibited drug resistance. The MAR ranged from 0.2 to 0.933, with an average of 0.567 (Table 3). Among the 26 examined isolates, 61.5% were MDR, 23.1% were XDR as detailed in Table 3.

**Table 3:** Antibiotic susceptibility of 26 *P. aeruginosa* (%) isolated from fish and fish handlers.

Antibiotic class	Antibiotic (µg/mL)	Resistant	Intermediate	Susceptible
Aminoglycosides	TOB (10 µg)	26 (100)	0 (0.0)	0 (0.0)
	CN (10 µg)	26 (100)	0 (0.0)	0 (0.0)
	AK (30 µg)	22 (84.6)	0 (0.0)	4 (15.4)
Carbapenems	MEM (10 µg)	6 (23.1)	2 (7.7)	18 (69.2)
	DOR (10 µg)	6 (23.1)	1 (3.8)	19 (73.1)
	IPM (10 µg)	1 (3.8)	2 (7.7)	23 (88.5)
Cephalosporins	CAZ (30 µg)	19 (73.1)	0 (0.0)	7 (26.9)
	FEP (30 µg)	9 (34.6)	0 (0.0)	17 (65.4)
Fluoroquinolones	LEV (5 µg)	11 (42.3)	2 (7.7)	13 (50.0)
	CIP (5 µg)	8 (30.7)	1 (3.8)	17 (65.4)
Lipopeptides	CT (25 µg)	26 (100)	0 (0.0)	0 (0.0)
Penicillins	PRL (30 µg)	10 (38.5)	2 (7.7)	14 (53.8)
	AMP (10 µg)	10 (38.5)	16 (61.5)	0 (00.0)
Monobactams	ATM (30 µg)	5 (19.2)	0 (0.0)	21 (80.8)
β-Lactam-β-lactamase-inhibitor combination	AMC (30 µg)	14 (53.8)	12 (46.2)	0 (00.0)

### Biofilm formation of *P. aeruginosa* isolates

All *P. aeruginosa* isolates (*n* = 26) exhibited biofilm-forming abilities, with 73.1% of the isolates exhibiting strong biofilm formation (Table 2). However, the remaining isolates exhibited moderate biofilm formation abilities (15.4%) and weak biofilm formation abilities (11.5%).

### Molecular characterization of*P. aeruginosa* isolates

The 16 S *rDNA* gene was molecularly identified in 26 biochemically suspected *P. aeruginosa strains.* Four virulence-associated genes, *las*B, *tox*A, *exo*U, and *opr*L, were identified in the *P. aeruginosa* isolates (Table 2). The *las*B virulence gene and *Exo*U virulence gene were detected in all the examined isolates (100%), followed by the *opr*L virulence gene (88.5%) and the *Tox*A virulence gene (76.9%) (Table 2). Three biofilm genes, *psl*A, *peI*A, and *las*R, were identified among the *P. aeruginosa* isolates (Table 2). Most of the biofilm genes were identified in *P. aeruginosa* isolates, with a high frequency in *psl*A (96.2%). The recovered *P. aeruginosa* isolates possessed the *las*I and *rhl*R QS genes in a prevalence of 84.6% and 61.5%, respectively.

### Sequence analysis of XDR *P. aeruginosa*

**Fig. 2:**
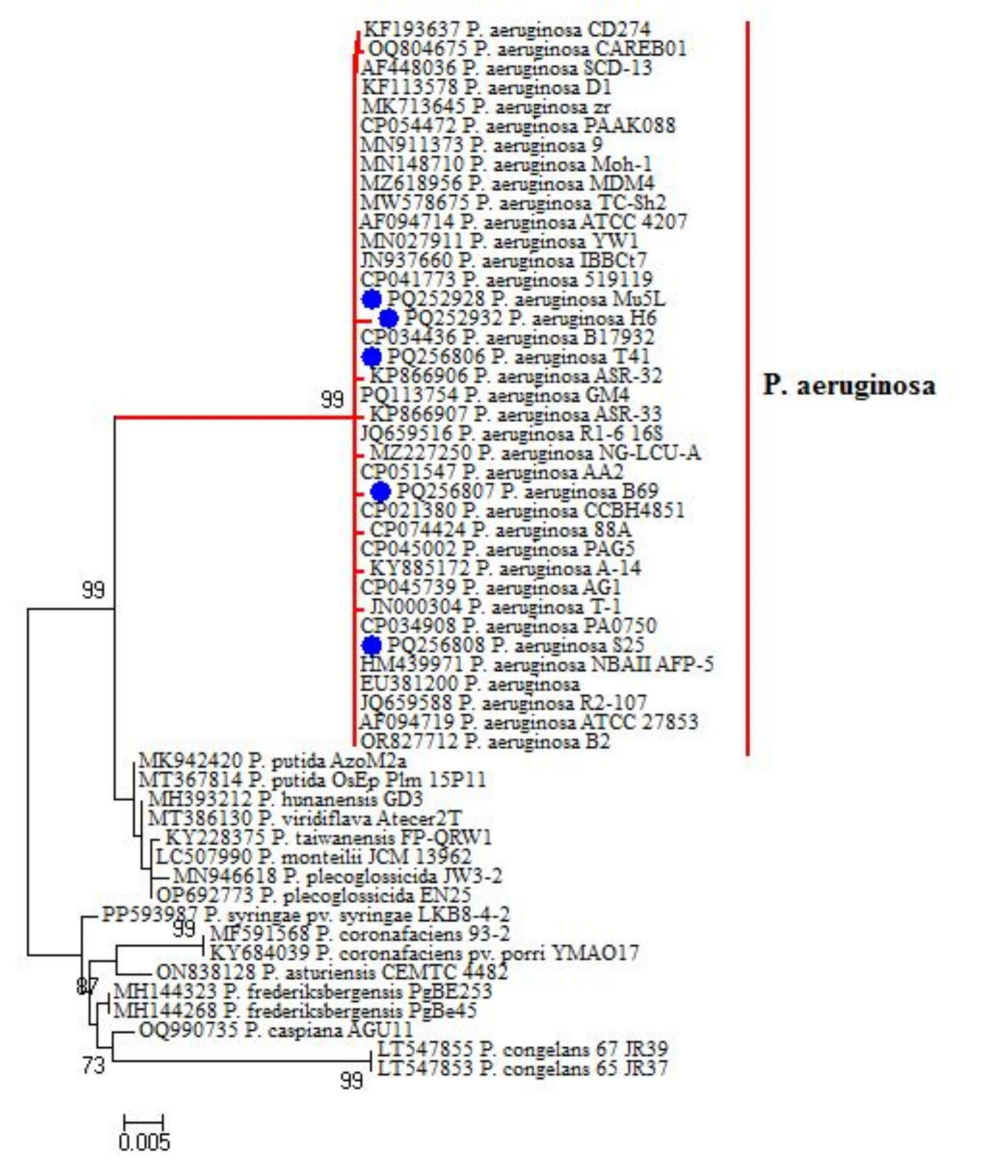
Phylogenetic tree of *P. aeruginosa* isolated from grey mullet, fish handlers, Nile tilapia, striped red mullet and Mediterranean mackerel (red dot) based on *16 S rDNA* gene sequences. A phylogenetic tree was generated using the neighbor-joining approach and 1000 bootstrap values.

The sequence analysis proved that the tested *P. aeruginosa* isolates in this study showed high genetic identity ranged from 99 to 100% with other *P. aeruginosa* strains isolated from different origins and geographical areas **(**Fig. 2**)**. From the alignment profile, *P. aeruginosa* isolate under accession number PQ252928 showed high genetic identity (100%) with other *P. aeruginosa* strains under accession numbers CP034436 and EU381200. The tested *P. aeruginosa* isolates under accession numbers PQ256806 and PQ256808 showed high genetic identity (100%) with *P. aeruginosa* strain under accession number OR827712. Furthermore, the tested *P. aeruginosa* isolate under accession number PQ256807 showed high genetic identity (99.9%) with *P. aeruginosa* strain under accession number OR827712. Besides, *P. aeruginosa* strain under accession number PQ252932 proved 99.3% genetic identity with *P. aeruginosa* strain under accession numbers HM439971, CP034436, and EU381200 (Fig. 2).

### Pathogenicity testing

**Fig. 3:**
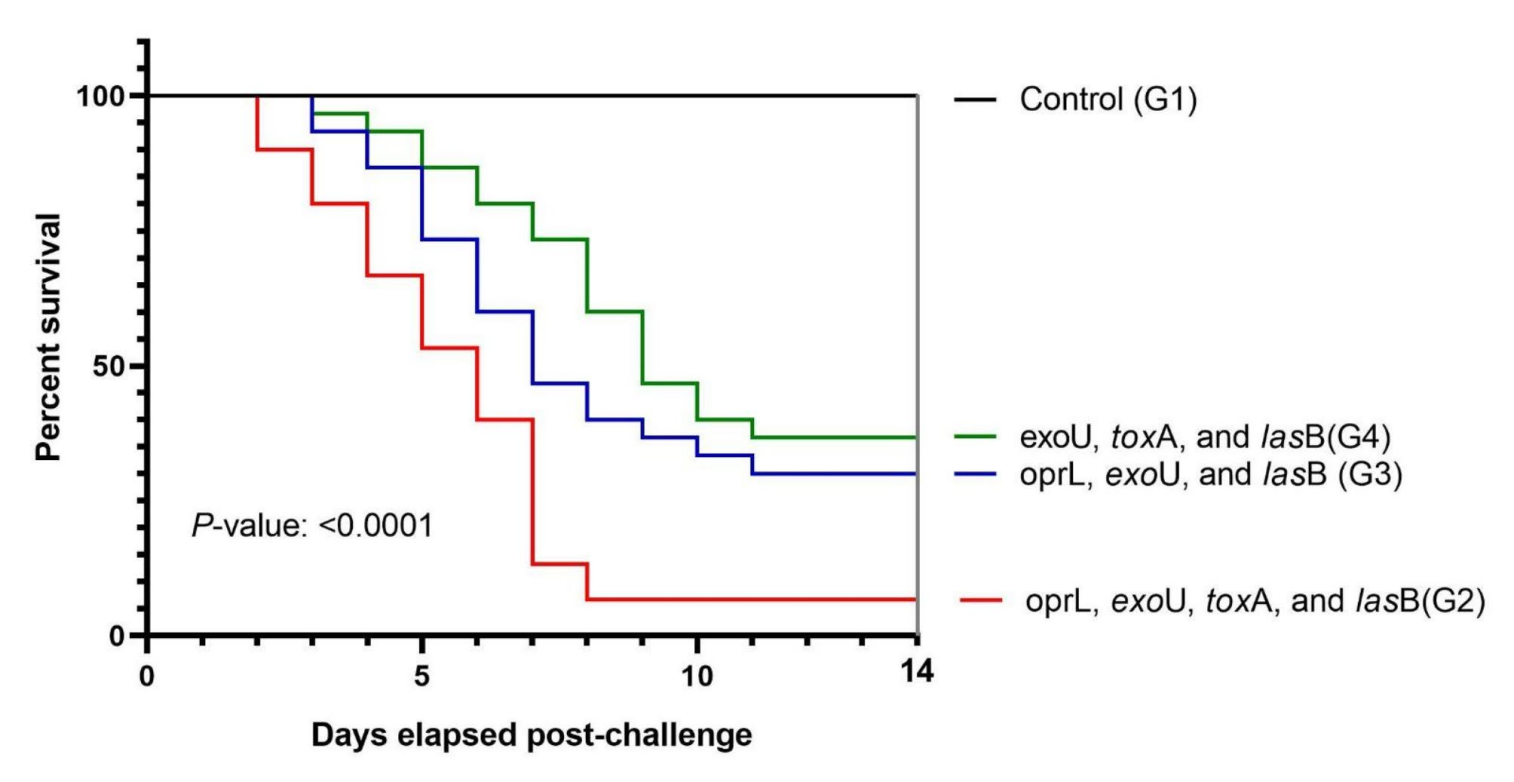
Kaplan-Meier survival plot showing the survivability of Nile tilapia over a 14-days challenge with *P. aeruginosa* that have various variable virulence potential. Different curves indicate the percent survival of fish (*n* = 30 per group). Fish in the control group were inoculated with PBS and fish within other groups were inoculated with *P. aeruginosa* harboring combination of virulence genes. A significant P-value indicates the significance of differences between all survival curves.

The clinical signs, postmortem lesions and fish mortality were recorded for 14 days after the challenge in all experimental groups. Clinical examination for the most of experimentally infected fish revealed superficial hemorrhages, detached scales and erosion of fins, while the postmortem findings showed typical septicemic signs manifested by congested kidney and liver, serous bloody fluid filling the abdominal cavity, enlarged spleen and necrotic gills (Figure S 1). It was noted that mortality rate was associated relatively with encoded virulence genes, where no mortalities or pathological lesions observed on fish of the control group while the maximum rate of mortality (93.3%) was recorded in fish group challenged with *P. aeruginosa* isolate harboring 4 virulence genes (*opr*L, *exo*U, *tox*A, and lasB) followed in descending order by isolates containing *opr*L, *exo*U, and *las*B genes (70%) and *exo*U, *tox*A, and *las*B (63.3%). The survivability of challenged fish groups with variable isolates was significantly different (P-value < 0.0001) (Fig. 3). Cumulative mortality in *O. niloticus* infected with a virulent strain M6 reached the peak on day 7 of the challenge, being of a shorter time compared to challenged groups with strain N1 and K10 that showed delayed mortality up to 11 days after inoculation.

### Hierarchical clustering (HC) of *P. aeruginosa* isolates

**Fig. 4:**
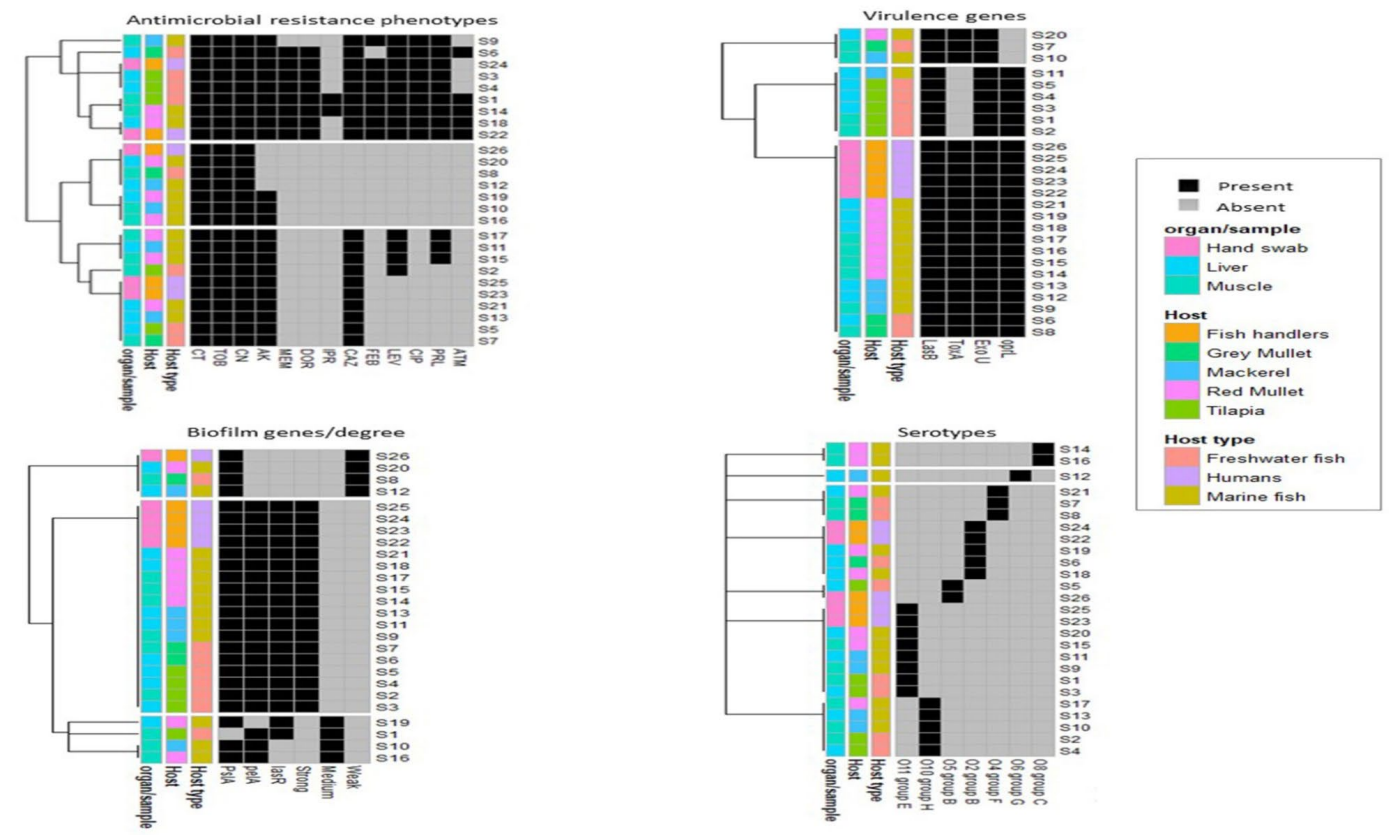
Heatmap supported by hierarchical clustering (dendrogram) showing the similarities among isolates from various hosts and organs based on the profile of each of the studied feature in the studied *P. aeruginosa* isolates. Black features. Annotation of isolates in relation to their hosts, host type and organs/samples are shown as color-coded categories.

**Fig. 5:**
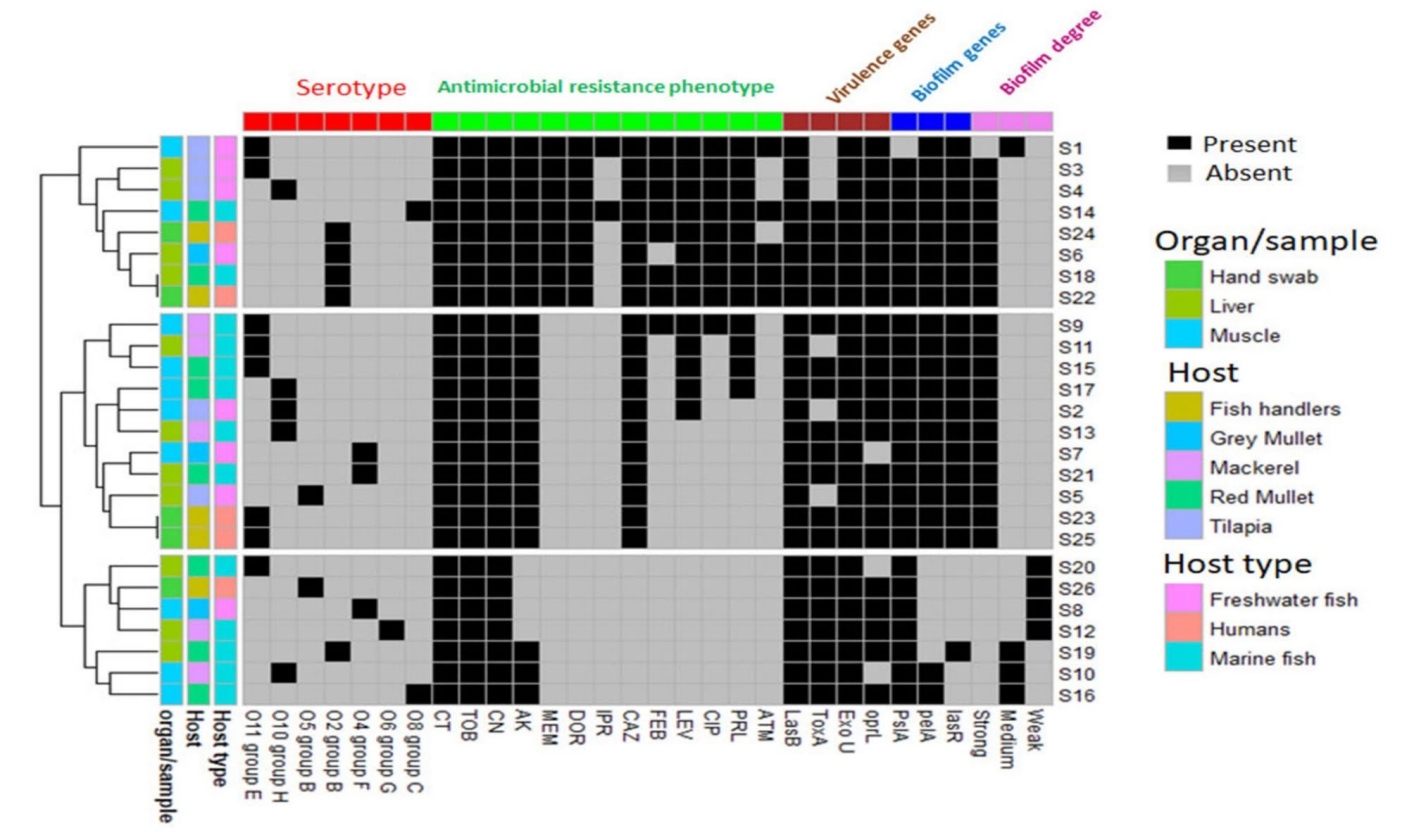
Heatmap supported by hierarchical clustering (dendrogram) showing the similarities among isolates from various hosts and organs based on a combined profile of features (i.e. antimicrobial resistance phenotypes, serotypes, virulence and biofilm genes/degrees) in the studied *P. aeruginosa* isolates. Black features. Annotation of isolates in relation to their hosts, host type and organs/samples are shown as color-coded categories.

**Fig. 6:**
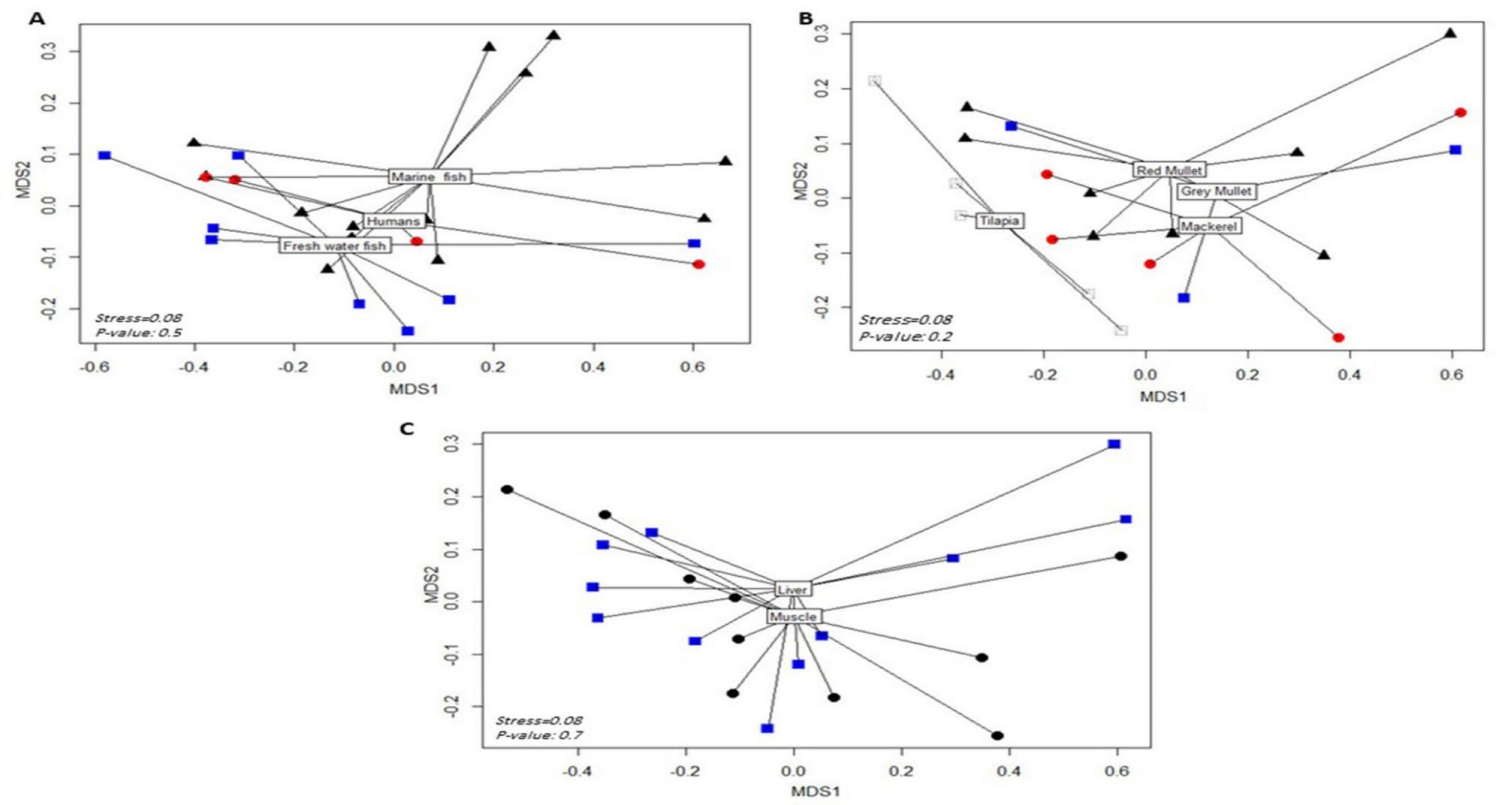
Non-metric multidimensional scaling plot showing the clustering of the isolates belonging to different hosts (**A**), fish types (**B**) and fish organs (**C**) based on the bray Curtis distance that were estimated among *P. aeruginosa* isolates.The center of each cluster are the centroid. The P-value represents the significance differences among clusters based on PERMANOVA test.

We ran (HC) on the isolates using the profile of each of the analyzed features (Fig. 4) and when all the features were combined (Fig. 5). As shown in Fig. 4, the AMR profile separated the 26 isolates into 7 clusters. The profiles of virulence genes and biofilm genes/degrees separated the isolates into 3 clusters, and the serotype profiles separated the isolates into 6 clusters. Although each of these clusters contained identical isolates, none of the isolates within each of the clusters belonged to the same host or sample type. As shown in Fig. 5, running the HCs on the isolates considering a combined profile of all features revealed the presence of 3 main clusters, with only 2 subclusters having identical isolates. One of these 2 subclusters included 2 human isolates, and the other cluster included a mixture of human and marine fish isolates. We then used the isolate features to obtain the Bray‒Curtis (Sorensen) distance, which is considered a dissimilarity indicator, and to generate the nMDS plot (Fig. 6). The nMDS plot showed that human, fresh fish and marine water fish isolates were not significantly separated (P value = 0.5) from fresh and that marine water fish lying further away from each other than human isolates were (Fig. 6A). Isolates from different types of fish were not significantly separated (P value = 0.2), with tilapia isolates exhibiting slight separation from other fish isolates, whereas isolates from gray mullet, red mullet, and mackerel overlapped (Fig. 6B). Liver and muscle isolates from all the fish were not significantly different (P value = 0.7) (Fig. 6C).

#### Using machine learning to discriminate isolates from different hosts

**Fig. 7:**
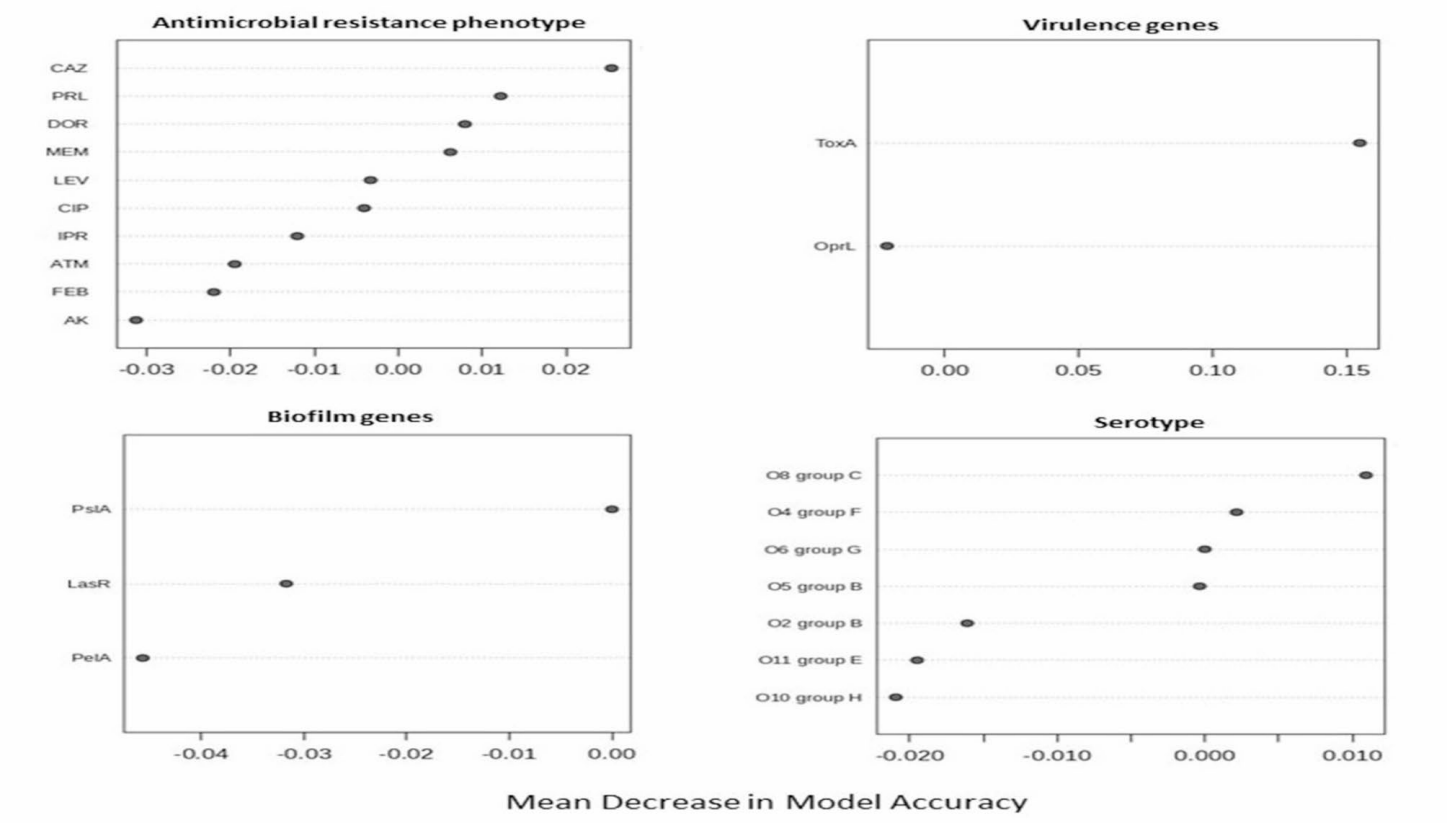
Random forest classification model showing the ranked significance (in a descending order) of various features they discriminate *P. aeruginosa* isolates from various hosts (i.e. humans, fresh water fish and marine fish). The discriminatory significance of a feature is shown as “mean decrease in accuracy”.

The ranked significance analyses of the random forest classification model (Fig. 7) revealed that phenotypic resistance to CAZ, the presence of *tox*A and *psl*A genes, and the O8 group C serotype were the features with the greatest dissimilarity among isolates from humans, freshwater fish, and marine fish. On the other hand, phenotypic resistance to AK, the presence of the *Opr*L gene, the *pel*A gene, and the O10 group H serotype exhibited the most similar profiles among these isolates.

#### Correlations between antimicrobial resistance (AMR) phenotype, virulence and biofilm-related genes

**Fig. 8:**
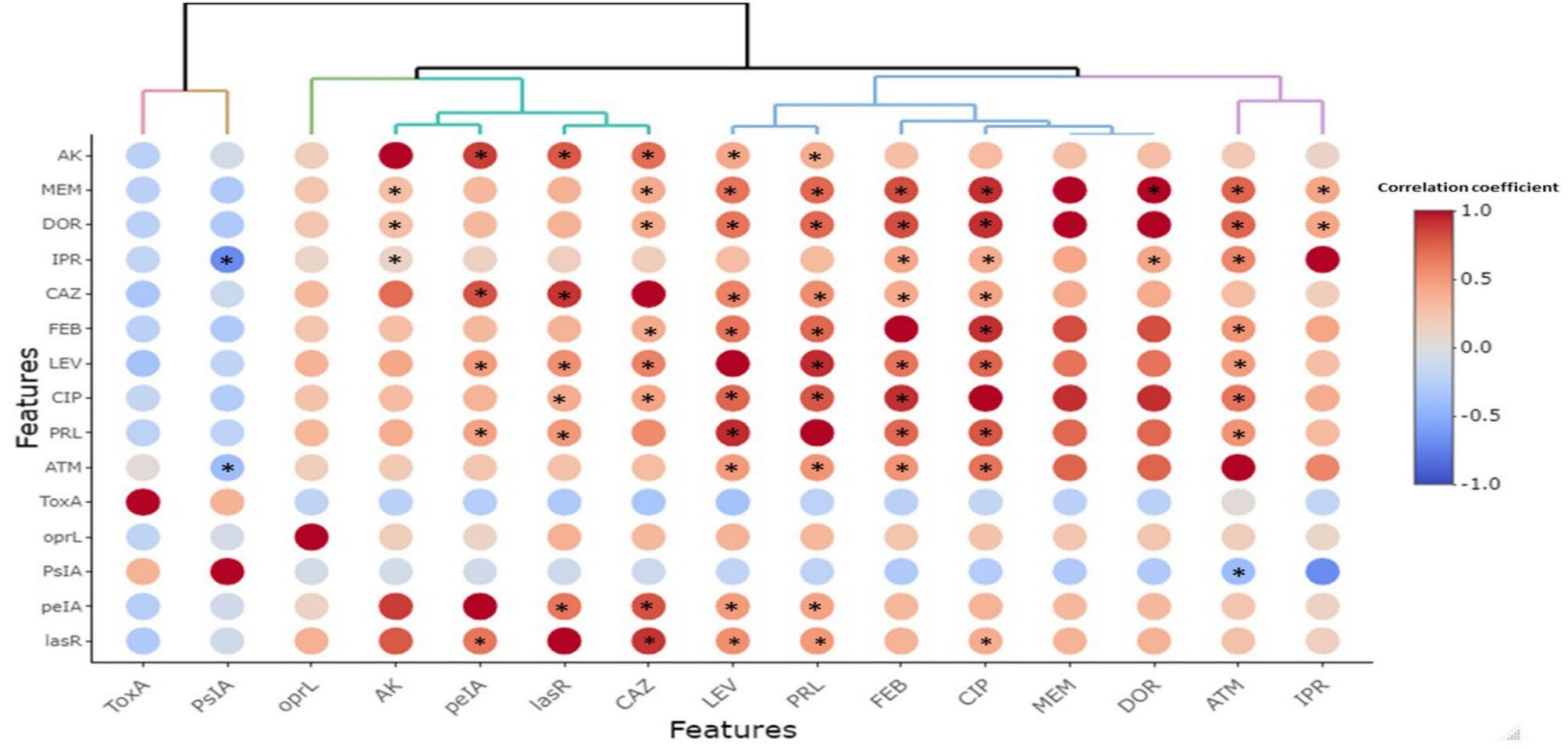
Pairwise correlation (*R*) among various *P. aeruginosa* isolates. Red and blue colors indicate positive and negative correlation, respectively on a scale of correlation coefficient (R) that ranges from +1 (positive) to −1 (negative). More intense colors imply stronger positive or negative correlations. Stars indicate significant correlation (at any level of P-value that are less than or equal to 0.05). Hierarchical clustering of the features based on the correlation values is shown as a dendrogram. Variables that are identical among all strains were excluded from this analysis and thus are not shown in the figure.

The correlation coefficient (R) and its significance (p values) are shown in Fig. 8. Among all the isolates (*n* = 26), all the AMR phenotypes to all the antibiotics were positively correlated with one another, with the most significant correlation being observed between the MEM and DOR (*R* = 1, P value < 0.001). This was followed by a slightly lower positive correlation between CIP and MEM and between DOR and FEB (*R* = 0.9, P values < 0.01). We observed a nonsignificant negative or weak positive correlation among most of the virulence genes. For the biofilm genes, significant positive correlations were found between *pelA* and *lasR* (P value = 0.0002). There was a nonsignificant correlation between any of the virulence and biofilm genes. The same is true for the correlation between AMR phenotypes and virulence genes. Interestingly, the *pelA* and *lasR* genes in the biofilm showed a strong positive correlation (*R* > 0.8) with phenotypic resistance to AK and CAZ and a moderate positive correlation (*R* = 0.5) with phenotypic resistance to the antibiotics LEV and PRL. The degree and significance of the previously mentioned correlations tended to be the same or slightly greater when the same analyses were performed on the isolates from each host.

## Discussion

*Pseudomonas aeruginosa* is a dangerous pathogen for public health, causes a variety of infections in humans, fish and animals, thus the identification of such pathogen should be considered in *Pseudomonas* studies^10^. In this study, *Pseudomonas* spp. were identified in 57.9% of the total examined samples, 49.6% and 8.3% of which originated from total fish samples and human samples, respectively, as detailed in Table S 2 and Table 1. These findings aligned with those of Abd-El-Maogoud et al.^48^, who isolated *Pseudomonas* spp. from frozen mackerel, frozen Saurus and tilapia in Egypt.

Table S 2 shows the higher frequency of *Pseudomonas* spp. in the fish liver samples. The liver was the most contaminated organ for *Pseudomonas* spp. infection, in contrast to the muscle in this study. These findings were consistent with the findings of Eissa et al.^49^, Abd ElTawab et al.^5^, and Algammal et al.^12^, who reported high contamination of *Pseudomonas* spp. in the liver. Among the identified *Pseudomonas* spp., *P. fluorescens* (34.4%) and *P. aeruginosa* (16.3%) were the most prevalent species in this study. Variation in the occurrence could be due to different geographical area, environment factors, fish species, host immunity state, and seasonal factor Algammal et al.^42^. The prevelance of *P. aeruginosa* in Nile tilapia and Golden grey mullet was 15.6% and 8.8%, respectively, while the respective prevalence in Striped red mullet and mackerel was 17.2% and 19.1%. *Pseudomonas aeruginosa* was isolated in freshwater fish (29%) and (32%) by Mohamed et al.^50^ and El-Tarabili et al.^51^, while Shahrokhi et al.^52^ reported a lower prevalence in freshwater fish (5%). Abd El Magaogoud et al.^48^ identified *P. aeruginosa* at 33%, 30%, and 23% prevalence in frozen mackerel, frozen Saurus and tilapia samples, respectively, in Egypt. In addition, Benie et al.^4^ reported incidences of 33.1% and 20% *P. aeruginosa* in freshwater fish and smoked fish, respectively. *Pseudomonas aeruginosa* was isolated in 46% of fish handlers in this study, which is consistent with Abdelraheem et al.^53^ who showed a prevalence of *P. aeruginosa* (45%) among patients with burn and surgical infected wounds in Egypt. The difference in the prevalence of *P. aeruginosa* can be attributed to differences in susceptibility to infection among different fish species^54^; to the ability of fish to tolerate variable degrees of salinity, temperature and pH; to persist in the environment, even in the absence of nutrients^55^; and to differences in hygienic measures during the capture, handling, and storage of fish^56^.

Among the *P. aeruginosa* isolates, seven different serotypes were identified, and serotype O11 belonging to group E was the predominant serotype (30.7%), consistent with previous findings of Benie et al.^4^ and Darwish et al.^57^, who identified the prevalence of serotype O11.

The prolonged use of antimicrobial agents for the treatment of *Pseudomonas* infection results in the generation of multidrug-resistant strains in aquatic ecosystems due to the transfer of R-plasmids^48^. *Pseudomonas aeruginosa* exhibits resistance to a variety of antibiotics, such as aminoglycosides, quinolones and β-lactams^58^. Generally, *Pseudomonas aeruginosa* can counter antibiotic attack by major intrinsic and acquired resistance mechanisms. The intrinsic mechanisms of *Pseudomonas* resistance are low permeability of the outer membrane, expression of efflux pumps that expel antibiotics out of the cell and the production of antibiotic inactivating enzymes. The acquired mechanisms of *Pseudomonas* resistance are resistance genes’ horizontal transfer or cell mutation^59^. In this study, *P. aeruginosa* isolates exhibited increased susceptibility to IPM (88.46%) and ATM (80.76%), which was consistent with the findings of Mohamed et al.^50^ and inconsistent with the findings of Benie et al.^4^, who reported the primary resistance of *P. aeruginosa* isolates to imipenem and ciprofloxacin. All *P. aeruginosa* isolates in this study exhibited resistance to tobramycin (TOB), gentamicin (CN) and colistin (CL), which is similar to the findings of Akhi et al.^60^, who demonstrated the full resistance of *P. aeruginosa* isolates to colistin. This resistance assists *P. aeruginosa* in nosocomial infection, food poisoning, and biofilm formation^61^. A study by Abd El-Baky et al.^62^ revealed the complete resistance of *P. aeruginosa* isolates to amoxicillin/ clavulanic acid and high resistance to ampicillin/sulbactam (68%), ceftazidime (63%) and azetreonam (60%). This contradiction with our results could be due to the method used, sample type, and sample size^60^. Drug resistance *Pseudomonas* mostly appeared in *P. aeruginosa* isolates (84.6%) in this study (Table 2), with a MAR value above 0.2 indicating the survival capacity of *P. aeruginosa* strains in a contaminated environment, and this greater defilement seems to result from the presence of numerous antimicrobial residues in the examined samples^63^. A MAR above 0.2 in this study was consistent with the findings of Darwish et al.^57^ in Egypt. Of tested isolates, 61.5% exhibited MDR and 23.1% of the tested isolates exhibited XDR in this study. *Peudomonas aeruginosa* isolates were MDR to 5 to 8 antimicrobial agents in four classes, 8 to 9 antimicrobial agents in six classes, and 12 antimicrobial agents in seven classes, besides, and were XDR to 11 to 13 antimicrobial agents in eight antimicrobial classes, 13 antimicrobial agents in seven classes, and 11 antimicrobial agent in 6 classes. However, Algammal et al.^12^ and El-Tarabili et al.^51^reported that the majorities of *P. aeruginosa* isolates were XDR to seven or eight antimicrobial classes. The prevalence of MDR *P. aeruginosa* varies from low (0–7.3%) in Saudi Arabia to high (50–75%) in Egypt due to heterogeneity in sample size and method of data collection^64^. PDR *Pseudomonas* was not detected in this study which was in consistent with Abd El-Baky et al.^62^. This study highlights the emergence of multiple drug-resistant *P. aeruginosa* strains in fish samples with public health problems; therefore, good hygienic practices; appropriate handling, storage and transportation; and surveillance systems for antimicrobial drugs are essential for preventing food poisoning associated with fish consumption^65^.

Biofilm formation results in a microbial community that affects the persistence of bacterial infections and reduces the sensitivity of bacteria to antibiotics through the attachment of many planktonic cells to extracellular polymeric substances^66^. In this study, all *P. aeruginosa* isolates exhibited biofilm-forming abilities, with 19 (73.1%) isolates exhibiting strong biofilm formation, which is consistent with El-Tarabili et al.^51^ who reported that 87.5% of *P. aeruginosa* isolates were biofilm producers. On the other hand, this study was inconsistent with the findings of El-Sapagh et al.^67^, who reported that 17.39% of *P. aeruginosa* isolates were strongly formed biofilms in Kufr El-Sheikh Governorate, Egypt. The moderate biofilm-forming and weak biofilm-forming capacities of the *P. aeruginosa* isolates were low in this study (15.4% each), which is inconsistent with the findings of Samie et al.^68^ and Abdulhaq et al.^69^, who reported more moderate biofilm-forming *P. aeruginosa* isolates than strong biofilm-forming *P. aeruginosa* isolates. The rates of variation in the biofilm-forming capacity of *P. aeruginosa* in this study compared with those in other previous investigations may be due to the difference in the number of *P. aeruginosa* isolates examined from different sources and localities^70^.

The diversity of virulence factors in *P. aeruginosa* is responsible for its pathogenicity in the host. The *las*B gene increases IL-8 production and decreases the innate immune response immunoglobulins, and complement compounds^71,72^. However, the *Exo*U gene is cytotoxic and acts as a marker for invasive *P. aeruginosa*^71^. In this study, *Las*B and *Exo*U virulence-associated genes were identified in all *P. aeruginosa* isolates, while the *opr*L and *tox*A genes were mostly identified (Table 2). In line with this study, Darwish et al.^57^ reported the full prevalence of the *Las*B gene in *P. aeruginosa* isolates from marine fish samples, whereas Farag et al.^73^ and Suresh et al.^74^ isolated *opr*L *and tox*A from most *P. aeruginosa* isolates from the examined fish. In studies by Algammal et al.^12^ reported that all *P. aeruginosa* isolates harbored *opr*L and *tox*A gene in the examined fish and El-Tarabili et al.^51^ revealed that all *P. aeruginosa* isolates harboured *opr*L gene and most isolates were positive for the *tox*A (87.1%) and *pel*A genes (84.3%). Consistent with our results, Benie et al.^4^ and Roy et al.^75^ reported the absence of the *Exo*U gene in *P. aeruginosa* isolates from examined fish. The variation in the frequency of virulence genes among these studies may be attributed to differences in the source of the isolates, the host, the geographic location, the number of isolates studied, the variation in sample types, degree of contamination, individual’s immune status and the ability of some *P. aeruginosa* strains to adapt to the unique circumstances present in enticing locations^76^. The *las*R and *pel*A biofilm genes have role in the formation of the carbohydrate-rich structure of the biofilm matrix, enhance resistance to aminoglycoside antibiotics in the biofim population, and the up-regulation and production of virulence determinants^15,53^. The *psy*A, *peI*A, and *las*R biofilm genes were mostly identified in *P. aeruginosa* isolates, with a high frequency in *psl*A (96.2%) in this study. All *P. aeruginosa* isolates were biofilm producers, consistent with the presence of biofilm-associated genes in this study. A previous study in India by Suresh et al.^74^ reported the prevalence of the *psl*A gene in 91.5% of *P. aeruginosa* isolates. Moreover, Ramazani et al.^77^ reported frequencies of 65.5% and 37.9% for the *psl*A and *pel*A genes, respectively.

In this study, the majority of *P. aeruginosa* isolates had the *las*I and *rhl*R QS genes. Consistent with our results, Algammal et al.^12^ and Sabharwal^78^ reported the presence of the *las*I and *rhl*R QS genes in the examined *P. aeruginosa* isolates. The *las* and *rhl* QS genes have role in the expression of virulence-related genes, antimicrobial resistance, and biofilm formation in *P. aeruginosa*. Quorum Sensing molecules are regulated by the *las* and *rhl* genes^79,80^. The differences in bacterial origins, genetic characteristics, isolation sources, and environmental conditions could also explain the differences observed between the present study and other previous investigations^81^.

In the present study, all retrieved isolates of *P. aeruginosa* from fish and human tested positive for the *16 S rDNA* gene. Likewise, the *16 S rDNA* sequence analyses emphasized that the tested *P. aeruginosa* strains were associated with the lineage of other *P. aeruginosa* strains from GeneBank originating from various sources and areas; accentuating the public health impact of *P. aeruginosa*.

Regarding the pathogenicity of *P. aeruginosa* in fish, the reported characteristic lesions of *P. aeruginosa* infection in this study were likewise with Derwa et al.^82^ demonstrated that *O .niloticus* challenged with *P. aeruginosa* showed clinical and postmortem lesions including external hemorrhages, loss of scales, fins erosion, necrotic gills, enlargement, and congestion of liver, kidney and spleen. Mortality rates showed direct relation to the encoded virulence genes as the obtained results matched with Algammal et al.^42^ who recorded the highest mortality rate (87.5%) in Nile tilapia infected with *P. aeruginosa* isolate encoding most of virulent genes and also reported the importance of both *opr*L and *tox*A virulence genes in the pathogenicity of *P. aeruginosa* strains. Our findings were also consistent with the results reported by Ghosh et al.^83^. who recorded similar pathological lesions of highly virulent *P. aeruginosa* strain as well over 90% mortality rates on infected fish.

In the present study, we utilized the power of multivariate statistics (particularly HC and nMD analyses) to obtain deeper insights into the diversity and colonicity of *P. aeruginosa* within hosts or samples. Our group has successfully applied these analyses to characterize *Aeromonas hydrophila*^84^, and *Salmonella* spp^85^. in various hosts, samples and localities. The HC analyses applied herein showed that none of the formed clusters contained isolates that belonged to the same host or sample, albeit all had identical profiles. This was also visualized by the nMD plot, where isolates from various hosts, fish, and samples considerably overlapped. This initially suggested increased heterogeneity of *P. aeruginosa* and a lack of adaptability in certain hosts or infection niches. This also indicates that the host or the infection site is not a driving factor for *P. aeruginosa* heterogeneity. Previous studies on these bacteria in Egypt identified various profiles of fitness traits, but they did not link this to the heterogeneity of the isolates at the host or sample level. Our study thus represents the first study to uncover these overlooked aspects of *P. aeruginosa* in Egypt. To answer the question of which of the analyzed features would best discriminate isolates from different hosts, we ran a random forest classification model, which best suits this task, as shown previously^84^. The observation that phenotypic resistance to CAZ, the presence of *tox*A and *psl*A genes and the O8 group C serotype were the most dissimilar features among the analyzed hosts suggested their usefulness in determining the host or sample origin of a particular isolate and hence could be used to track the source of infection during outbreaks. To generalize these data, additional wide-scale analyses need to be performed. The survival of bacteria is a reflection of the co-occurrence of various fitness traits, which are usually correlated^86^. The high positive correlation between the MEM and DOR is likely due to their shared evolution, as they belong to the same antimicrobial group, carbapenems. The high correlation among biofilm genes observed in our study suggested that these genes might be carried on the same mobile genetic element or be present in the same location on the bacterial chromosome.

## Conclusion

In this study, *P. aeruginosa* was frequently prevalent *Pseudomonas* spp. isolated from Nile tilapia, Golden grey mullet, Mediterranean horse mackerel, Striped red mullet, and fish handlers at different retail fish markets in Damietta Governorate, Egypt. The recovery of MDR and XDR strains of *P. aeruginosa* indicate improper use of antibiotics. *Las*B and *Exo*U genes were the most prevalent virulence genes associated with *P. aeruginosa*.

IPM was considered the drug of choice in this study. All *P. aeruginosa* isolates were biofilm producers with a high prevalence of the *psl*A biofilm gene. The *las*I and *rhl*R QS genes were identified in majority of biofilm forming *P. aeruginosa* isolates. Therefore, good hygienic practices, appropriate handling during the storage and transportation of fish, and routine antimicrobial susceptibility testing should be considered to prevent the emergence of drug-resistant *P. aeruginosa* strains at risk of hazard public risk associated with fish consumption. To the best of our knowledge, this study is the first to apply multivariate statistics to evaluate the fitness features of *P. aeruginosa* isolated from multiple human and fish hosts in Damietta Governorate, Egypt. A further research that in plan is to expose the recovered isolates to whole genome sequencing and bioinformatics to reveal the antimicrobial resistance, biofilm and virulence genes at the genome-level. This will enable performing more focused pathogenicity experiments, and to assess the use of alternative natural antimicrobial agents and antibiofilm agents against strong biofilm-forming and MDR *P. aeruginosa* strains.

## Electronic supplementary material

Below is the link to the electronic supplementary material.

Supplementary Material 1

## Data Availability

The datasets for this study can be found in the article/ supplementary materials, further inquiries can be directed to the corresponding author.

## References

[1] Cardoso, J. C., Bergqvist, C. A., Felix, R. C. & Larhammar, D. Corticotropin-releasing hormone family evolution: five ancestral genes remain in some lineages. *J. Mol. Endocrinol.*** 57**, 73–86 (2016). 10.1530/JME-16-0051 27220618

[2] Morshdy, A. E. M. et al. Tetracycline residues in tilapia and catfish tissue and the effect of different cooking methods on oxytetracycline and doxycycline residues. *J. Consum. Prot. Food Saf.*** 17**, 387–393 (2022).

[3] Mhenni, N. B., Alberghini, G., Giaccone, V., Truant, A. & Catellani, P. Prevalence and antibiotic resistance phenotypes of *Pseudomonas* spp. in fresh fish fillets. *Foods ***12**, 950. 10.3390/foods12050950 (2023). 36900467 10.3390/foods12050950 PMC10000908

[4] Benie, C. K. et al. Characterization of virulence potential of *Pseudomonas aeruginosa* isolated from bovine meat, fresh fish, and smoked fish. *Eur. J. Microbiol. Immunol. (Bp) ***7**, 55–64. 10.1556/1886.2016.00039 (2016). 10.1556/1886.2016.00039 PMC5372481 28386471

[5] Tawab, A. E., Maarouf, A., Ahmed, N. & A. & Detection of virulence factors of *Pseudomonas* species isolated from fresh water fish by PCR. *Benha Vet. Med. J.*** 30**, 199–207 (2016).

[6] Morshdy, A. E., Hafez, A. E., Darwish, W. S., Hussein, M. A. & Tharwat, A. E. Heavy metal residues in canned fishes in Egypt. *Jpn. J. Vet. Res.*** 61**, S54–S57 (2013). 23631154

[7] Darwish, W. S., El-Ghareeb, W. R., Alsayeqh, A. F. & Morshdy, A. E. M. Foodborne intoxications and toxicoinfections in the Middle East. In* Food Safety in the Middle East*, 109–141 (Acad. Press, 2022).

[8] Gram, L. & Huss, H. H. Fresh and processed fish and shellfish. In *The Microbiological Safety and Quality of Food* (eds Lund, B. M. et al.) 472–506 (Aspen Publishers Inc., 2000).

[9] Tran, N. B. V. et al. Prevalence and virulence of commensal *Pseudomonas aeruginosa* isolates from healthy individuals in Southern Vietnam (2018–2020). *Biomedicines*** 11**, 54. 10.3390/biomedicines11010054 (2023). 10.3390/biomedicines11010054 PMC9855430 36672562

[10] Shahat, H., Mohamed, H., Al-Azeem, A., Nasef, S. & M. & Molecular detection of some virulence genes in *Pseudomonas aeruginosa* isolated from chicken embryos and broilers with regard to disinfectant resistance. *SVU-Intern. J. Vet. Sci. ***2**, 52–70. 10.21608/svu.2019.12365.1011 (2019).

[11] Kadmy, I. M. S. et al. The secrets of environmental *Pseudomonas aeruginosa* in slaughterhouses: Antibiogram profile, virulence, and antibiotic resistance genes.* Folia Microbiol (Praha)*. 10.1007/s12223-023-01116-1 (2023). 10.1007/s12223-023-01116-1 38091178

[12] Algammal, A. M. et al. *opr*L gene sequencing, resistance patterns, virulence genes, Quorum Sensing and antibiotic resistance genes of XDR *Pseudomonas aeruginosa* isolated from broiler chickens. *Infect. Drug Resist. ***13**, 853–867. 10.2147/IDR.S401473 (2023). 10.2147/IDR.S401473 PMC9937075 36818807

[13] Azam, M. W. & Khan, A. U. Updates on the pathogenicity status of *Pseudomonas aeruginosa*. *Drug Discov. Today*** 24**, 350–359. 10.1016/j.drudis.2018.07.003 (2019). 30036575 10.1016/j.drudis.2018.07.003

[14] Maita, P. & Boonbumrung, K. Association between biofilm formation of *Pseudomonas aeruginosa* clinical isolates versus antibiotic resistance and genes involved with biofilm. *J. Chem. Pharm. ***6**, 1022–1028 (2014).

[15] Heydari, S. & Eftekhar, F. Biofilm formation and β-lactamase production in burn isolates of* Pseudomonas aeruginosa*.* Jundishapur J. Microbiol.*** 8**, 1–5 (2015). 10.5812/jjm.15514 PMC4417555 25964848

[16] Park, W. S. et al. Benzyl isothiocyanate attenuates inflammasome activation in* Pseudomonas aeruginosa* LPS-stimulated THP-1 cells and exerts regulation through the MAPKs/NF-kappaB pathway.* Int. J. Mol. Sci.*** 23**, 1–10 (2022). 10.3390/ijms23031228 PMC8835927 35163151

[17] Yang, J. J., Tsuei, K. C. & Shen, E. P. The role of type III secretion system in the pathogenesis of *Pseudomonas aeruginosa* microbial keratitis. *Tzu Chi Med. J.*** 34**, 8–14 (2022). 35233350 10.4103/tcmj.tcmj_47_21 PMC8830546

[18] Rezaloo, M., Motalebi, A., Mashak, Z. & Anvar, A. Prevalence, antimicrobial resistance, and molecular description of Pseudomonas aeruginosa isolated from meat and meat products. *J. Food Qual. *10.1155/2022/9899338 (2022).

[19] Marmont, S., Rich, D. & Whitney, C. Oligomeric lipoprotein *Pel*C guides *pel* polysaccharide export across the outer membrane of *Pseudomonas aeruginosa*. *PNAS ***114**, 2893–2897 (2017). 10.1073/pnas.1613606114 PMC5358405 28242707

[20] Finnan, S., Morrissey, J. P., O’Gara, F. & Boyd, E. F. Genome diversity of *Pseudomonas aeruginosa* isolates from cystic fibrosis patients and the hospital environment. *J. Clin. Microbiol.*** 42**, 5783–5792 (2004). 15583313 10.1128/JCM.42.12.5783-5792.2004 PMC535267

[21] ISO 6887-3. *Microbiology of the food chain-preparation of test Samples, Initial Suspension and Decimal Dilutions for Microbiological examination—Part 3* (Specific rules for the preparation of fish and fishery products, 2017).

[22] Austin, B. & Austin, D. Characteristics of the pathogens: Gram-negative bacteria. In *Bacterial Fish Pathogens. Diseases of Farmed and Wild Fish*, 81–150. (2007).

[23] ISO 13720. Meat and meat products—Enumeration of presumptive Pseudomonas spp. (2010).

[24] Cheesbrough, M. *District Laboratory Practice in Tropical Countries* 2nd edn, 178–187 (Cambridge University Press, 2006).

[25] Glupczynski, Y. et al. Detection and characterization of class A extended-spectrumb-lactamase-producing *Pseudomonas aeruginosa* isolates in Belgian hospitals. *J. Antimicrob. Chemother.*** 65**, 866–871 (1982). 10.1093/jac/dkq048 20200037

[26] Legakis, N., Majtan, V. & Wang, S. Serotypes of *Pseudomonas aeruginosa* in clinical specimens in relation to antibiotic susceptibility. *J. Clin. Microbiol.*** 16**, 458–463 (1982). 6813351 10.1128/jcm.16.3.458-463.1982 PMC272389

[27] Spilker, T., Coenye, T., Vandamme, P. & LiPuma, J. J. PCR-based assay for differentiation of *Pseudomonas aeruginosa* from other *Pseudomonas* species recovered from cystic fibrosis patients. *J. Clin. Microbiol.*** 42**, 2074–2079 (2004). 15131172 10.1128/JCM.42.5.2074-2079.2004 PMC404678

[28] Clinical and Laboratory Standards Institute (CLSI). Performance standards for antimicrobial susceptibility testing. 31th ed. Supplement M100 (2021). Clinical and Laboratory Standards Institute: Wayne, PA, USA.

[29] Krumperman, P. H. Multiple antibiotic resistance indexing of *Escherichia coli* to identify high-risk sources of fecal contamination of foods. *Appl. Environ. Microbiol. ***46**, 165–170 (1983). 6351743 10.1128/aem.46.1.165-170.1983 PMC239283

[30] Magiorakos, A. P. et al. Multidrug-resistant, extensively drug-resistant and pandrug-resistant bacteria: an international expert proposal for interim standard definitions for acquired resistance. *Clin. Microbiol. Infect. ***18**, 268–281. 10.1111/j.1469-0691.2011.03570.x (2012). 21793988 10.1111/j.1469-0691.2011.03570.x

[31] Kırmusaoğlu, S. in *The Methods for Detection of Biofilm and Screening Antibiofilm Activity of Agents in Antimicrobials, Antibiotic Resistance, Antibiofilm Strategies and Activity Methods* (ed Kırmusaoğlu, S.) 1–17 (IntechOpen, 2019).

[32] Saxena, S., Banerjee, G., Garg, R. & Singh, M. Comparative study of biofilm formation in Pseudomonas aeruginosa isolates from patients of lower respiratory tract infection. *J. Clin. Diagn. Res.*** 8**, DC09–DC11. 10.7860/JCDR/2014/7808.4330 (2014). 24995174 10.7860/JCDR/2014/7808.4330 PMC4079995

[33] Matar, G. M., Ramlawi, F., Hijazi, N., Khneisser, I. & Abdelnoor, A. M. Transcription levels of *Pseudomonas aeruginosa* Exotoxin a gene and severity of symptoms in patients with otitis externa. *Curr. Microbiol. ***45**, 350–354 (2002). 12232666 10.1007/s00284-002-3703-z

[34] Winstanley, C. et al. Genotypic and phenotypic characteristics of *Pseudomonas aeruginosa* isolates associated with ulcerative keratitis. *J. Med. Microbiol. ***54**, 519–526 (2005). 15888458 10.1099/jmm.0.46005-0

[35] Xu, J., Moore, J. E., Murphy, P. G., Millar, B. C. & Elborn, J. S. Early detection of *Pseudomonas aeruginosa*–comparison of conventional versus molecular (PCR) detection directly from adult patients with cystic fibrosis (CF). *Ann. Clin. Microbiol. Antimicrob.*** 3**, 21 (2004). 15496232 10.1186/1476-0711-3-21 PMC529303

[36] Ghadaksaz, A., Fooladi, A. A. I., Hosseini, H. M. & Amin, M. The prevalence of some *Pseudomonas* virulence genes related to biofilm formation and alginate production among clinical isolates. *J. Appl. Biomed. ***13**, 61–68 (2015).

[37] Saleh, M. M., Sadeq, R. A., Latif, H. K. A., Abbas, H. A. & Askoura, M. Zinc oxide nanoparticles inhibits quorum sensing and virulence in *Pseudomonas aeruginosa*. *Afr. Health Sci. ***19**, 2043–2055 (2019). 31656488 10.4314/ahs.v19i2.28 PMC6794539

[38] Bratu, S., Gupta, J. & Quale, J. Expression of the las and rhl quorum-sensing systems in clinical isolates of Pseudomonas aeruginosa does not correlate with efflux pump expression or antimicrobial resistance. *J. Antimicrob. Chemother.*** 58**, 1250–1253 (2006). 17030516 10.1093/jac/dkl407

[39] Altschul, S. F., Gish, W., Miller, W., Myers, E. W. & Lipmanl, D. J. Basic local alignment search tool. *J. Mol. Biol.*** 215**, 403–410 (1990). 2231712 10.1016/S0022-2836(05)80360-2

[40] Thompson, J. D., Higgins, D. G. & Gibson, T. J. CLUSTAL W: improving the sensitivity of progressive multiple sequence alignment through sequence weighting, position-specific gap penalties and weight matrix choice. *Nucleic Acids Res.*** 22**, 4673–4680 (1994). 7984417 10.1093/nar/22.22.4673 PMC308517

[41] Tamura, K., Stecher, G., Peterson, D., Filipski, A. & Kumar, S. MEGA6: molecular evolutionary genetics analysis version 6.0. *Mol. Biol. Evol.*** 30**, 2725–2729 (2013). 24132122 10.1093/molbev/mst197 PMC3840312

[42] Algammal, A. M. et al. Emerging MDR-*Pseudomonas aeruginosa* in fish commonly harbor *opr*L and *tox*A virulence genes and *bla*TEM, *bla*CTX-M, and *tet*A antibiotic-resistance genes. *Sci. Rep. ***10**, 15961. 10.1038/s41598-020-72264-4 (2020). 32994450 10.1038/s41598-020-72264-4 PMC7524749

[43] Kolde, R. Package pheatmap: Pretty heat map 1–8 (2019).

[44] Oksanen Vegan: Community Ecology Package. R software (2022).

[45] Anderson, M. J. Permutational Multivariate Analysis of Variance (PERMANOVA). Wiley StatsRef: Statistics Reference Online: 1–15 (2017).

[46] Lu, Y., Pang, Z. & Xia, J. Comprehensive investigation of pathway enrichment methods for functional interpretation of LC–MS global metabolomics data. *Brief. Bioinform.*** 24** (2022). 10.1093/bib/bbac553 PMC9851290 36572652

[47] Jr, F. E. H. Hmisc: Harrell Miscellaneous. R software (2023).

[48] Abd-El-Maogoud, H. A. E. N., Edris, A. B. M., Mahmoud, A. H. & Maky, M. A. Occurrence and characterization of *Pseudomonas* species isolated from fish marketed in Sohag governorate, Egypt. *SVU- Int. J. Vet. Sci.*** 4**, 76–84 (2021).

[49] Eissa, N., El-Ghiet, E., Shaheen, A. & Abbass, A. Characterization of Pseudomonas species isolated from tilapia Oreochromis Niloticus in Qaroun and Wadi-El-Rayan lakes, Egypt. *Glob. Vet.*** 5**, 116–121 (2010).

[50] Mohamed, E. A., Nawar, A. E. & Hegazy, E. E. Insight into quorum sensing genes lasr and rhlr, their related virulence factors and antibiotic resistance pattern in *Pseudomonas aeruginosa* isolated from ocular infections. *Microbes Infect. Dis. ***4**, 575–589. 10.21608/MID.2023.197968.1480 (2023).

[51] El-Tarabili, R. M., Eid, H. M., Elghayaty, H. A. A. & Zaghloul, E. M. Detection of *pel*A and associated virulence genes in emerging multidrug- and extensively drug-resistant (MDR and XDR) *Pseudomonas aeruginosa* isolated from *Oreochromis niloticus*. *Bulg. J. Vet. Med. ***26**, 524–541 (2023).

[52] Shahrokhi, G. R., Rahimi, E. & Shakerian, A. The prevalence rate, pattern of antibiotic resistance, and frequency of virulence factors of Pseudomonas aeruginosa strains isolated from fish in Iran. *J. Food Qual.*** 2022**, 8990912. 10.1155/2022/8990912 (2022).

[53] Abdelraheem, M. W., Abdelkader, E. A., Mohamed, S. E. & Mohammed, S. M. Detection of biofilm formation and assessment of biofilm genes expression in different *Pseudomonas aeruginosa* clinical isolates. *Meta Gene ***23**. 10.1016/j.mgene.2020.100646 (2020).

[54] Carol, G. R., Jeyasanta, K. I., Mani, A. E. & Patterson, J. Prevalence of *Pseudomonas* sp in fin fishes and their antibiotic susceptibility. *J. Pure Appl. Microbiol. ***7**, 677–681 (2013).

[55] Duman, M. et al. The diversity of *Pseudomonas* species isolated from fish farms in Turkey. *Aquaculture*** 535**, 736369 (2021).

[56] Salem, A. M., Osman, I. M. & Shehata, S. M. Assessment of psychrotrophic bacteria in frozen fish with special reference to *Pseudomonas* species. *Benha Vet. Med. J.*** 34**, 140–148 (2018).

[57] Darwish, W. S. et al. Prevalence of *Pseudomonas* spp. in marine water fish intended for human consumption. *J. Adv. Vet. Res. ***13**, 1147–1152 (2023).

[58] Hancock, R. E. & Brinkman, F. S. Function of *Pseudomonas* porins in uptake and efflux. *Annu. Rev. Microbiol. ***56**, 17–38 (2002). 12142471 10.1146/annurev.micro.56.012302.160310

[59] Breidenstein, E. B., de la Fuente-Nunez, C. & Hancock, R. E. *Pseudomonas aeruginosa*: all roads lead to resistance. *Trends Microbiol.*** 19**, 419–426 (2011). 21664819 10.1016/j.tim.2011.04.005

[60] Akhi, M. T. et al. Antibiotic susceptibility pattern of aerobic and anaerobic bacteria isolated from surgical site infection of hospitalized patients. *Jundishapur J. Microbiol. ***8**, e20309. 10.5812/jjm.20309v2 (2015). 26421133 10.5812/jjm.20309v2 PMC4584138

[61] Tremblay, Y. D., Hathroubi, S. & Jacques, M. Les biofilms bactériens: leur importance en santé animale et en santé publique [Bacterial biofilms: their importance in animal health and public health]. *Can. J. Vet. Res.*** 78**, 110–116 (2014). 24688172 PMC3962273

[62] Abd El-Baky, R. M. et al. Prevalence and some possible mechanisms of colistin resistance among multidrug-resistant and extensively drug-resistant *Pseudomonas aeruginosa*. *Infect. Drug Resist.*** 3**, 323–332. 10.2147/IDR.S238811 (2020). 10.2147/IDR.S238811 PMC7006860 32099423

[63] Talukder, A., Rahman, M. M., Chowdhury, M. M. H., Mobashshera, T. A. & Islam, N. N. Plasmid profiling of multiple antibiotic-resistant *Pseudomonas aeruginosa* isolated from soil of the industrial area in Chittagong, Bangladesh. *Beni-Suef Univ. J. Basic. Appl. Sci. ***10**, 44. 10.1186/s43088-021-00131-w (2021).

[64] Al-Orphaly, M. et al. Epidemiology of multidrug-resistant *Pseudomonas aeruginosa* in the Middle East and North Africa region. *mSphere* 6, e00202-21. 10.1128/mSphere.00202-21 (2021). 10.1128/mSphere.00202-21 PMC8265635 34011686

[65] Tiamiyu, A. M., Soladoye, M. O., Adegboyega, T. T. & Adetona, M. O. Occurrence and antibiotic sensitivity of bacterial strains isolated from Nile tilapia, *Oreochromis niloticus* obtained in Ibadan, Southwest Nigeria. *J. Biosci. Med. ***3**, 19–26. 10.4236/jbm.2015.35003 (2015).

[66] Hall-Stoodley, L., Costerton, J. W. & Stoodley, P. Bacterial biofilms: from the natural environment to infectious diseases. *Nat. Rev. Microbiol. ***2**, 95–108. 10.1038/nrmicro821 (2004). 10.1038/nrmicro821 15040259

[67] El-Sapagh, S., El-Shenody, R., Pereira, L. & Elshobary, M. Unveiling the potential of algal extracts as promising antibacterial and antibiofilm agents against multidrug-resistant *Pseudomonas aeruginosa*: in vitro and in silico studies including molecular docking. *Plants (Basel)*** 12**, 3324. 10.3390/plants12183324 (2023). 37765485 10.3390/plants12183324 PMC10537748

[68] Samie, S., Rizk, Y., Al-Husseini, N. & Ibrahim, D. Biofilm genes among drug resistant *Pseudomonas aeruginosa*. *Benha J. Appl. Sci.*** 5**, 211–223. 10.21608/bjas.2020.187211 (2020).

[69] Abdulhaq, N., Nawaz, Z. & Zahoor, M. A. Association of biofilm formation with multi drug resistance in clinical isolates of *Pseudomonas aeruginosa*. *EXCLI J.*** 19**, 201–208 (2020). 32256266 10.17179/excli2019-2049 PMC7105944

[70] Abdi-Ali, A., Hendiani, S., Mohammadi, P. & Gharavi, S. Assessment of biofilm formation and resistance to imipenem and ciprofloxacin among clinical isolates of *Acinetobacter baumannii* in Tehran. *Jundishapur J. Microbiol. ***7**, e8606. 10.5812/jjm.8606 (2014). 10.5812/jjm.8606 PMC4138664 25147652

[71] Hosu, M. C., Vasaikar, S. D., Okuthe, G. E. & Apalata, T. Detection of extended spectrum beta-lactamase genes in *Pseudomonas aeruginosa* isolated from patients in rural Eastern Cape Province, South Africa. *Sci. Rep. ***11**, 7110. 10.1038/s41598-021-86570-y (2021). 33782509 10.1038/s41598-021-86570-y PMC8007629

[72] Fadhil, L., Al-Marzoqi, A. H., Zahraa, M. A. & Shalan, A. A. Molecular and phenotypic study of virulence genes in a pathogenic strain of Pseudomonas aeruginosa isolated from various clinical origins by PCR: profiles of genes and toxins. *Res. J. Pharm. Biol. Chem. Sci. ***7**, 590–598 (2016).

[73] Farag, H. A. M., Tawab, A. E., Maarouf, A. A., Ahmed, W. & A. A. A. & Molecular detection of some virulence factors of *Pseudomonas aeruginosa* isolated from freshwater fishes at Qalubiya Governorate, Egypt. *Benha Vet. Med. J. ***43**, 80–84. https://bvmj.journals.ekb.eg (2023).

[74] Suresh, S. et al. Comparison of antibiofilm activity of *Pseudomonas aeruginosa* phages on isolates from wounds of diabetic and non-diabetic patients. *Microorganisms ***11**, 2230. 10.3390/microorganisms11092230 (2023). 37764074 10.3390/microorganisms11092230 PMC10536433

[75] Roy, P. H. et al. Complete genome sequence of the multiresistant taxonomic outlier *Pseudomonas aeruginosa* PA7. *PLoS One*** 5**, e8842. 10.1371/journal.pone.0008842 (2010). 10.1371/journal.pone.0008842 PMC2809737 20107499

[76] Aljebory, I. S. PCR detection of some virulence genes of *Pseudomonas aeruginosa* in Kirkukcity. *Iraq J. Pharm. Sci.*** 10**, 1068–1071 (2018).

[77] Ramazani, R., Izadi Amoli, R., Taghizadeh Armaki, M., Pournajaf, A. & Kaboosi, H. A. Molecular new update on the biofilm production and carbapenem resistance mechanisms in clinical Pseudomonas aeruginosa isolates. *Iran. J. Med. Microbiol.*** 16**, 557–565. http://ijmm.ir/article-1-1696-en.html (2022).

[78] Sabharwal, N., Dhall, S., Chhibber, S. & Harjai, K. Molecular detection of virulence genes as markers in *Pseudomonas aeruginosa* isolated from urinary tract infections. *Int. J. Mol. Epidemiol. Genet.*** 5**, 125 (2014). 25379131 PMC4214259

[79] Hartmann, A. & Schikora, A. Quorum sensing of bacteria and trans-kingdom interactions of N-acyl homoserine lactones with eukaryotes. *J. Chem. Ecol. ***38**, 704–713. 10.1007/s10886-012-0141-7 (2012). 22648507 10.1007/s10886-012-0141-7

[80] Khorvash, F., Yazdani, M., Shabani, S. & Soudi, A. *Pseudomonas aeruginosa*-producing metallo-β-lactamases (VIM, IMP, SME, and AIM) in the clinical isolates of intensive care units, a university hospital in Isfahan, Iran. *Adv. Biomed. Res. ***6**, 25 (2017). 29285477 10.4103/2277-9175.219412 PMC5735557

[81] Pajavand, H. et al. Evaluation of combined carbon dots and ciprofloxacin on the expression level of *psl*A, *pel*A, and *ppy*R genes and biofilm production in ciprofloxacin-resistant *Pseudomonas aeruginosa* isolates from burn wound infection in Iran. *J. Glob Antimicrob. Resist.*** 35**, 289–296. 10.1016/j.jgar.2023.10.005 (2023). 37844801 10.1016/j.jgar.2023.10.005

[82] Derwa, H., AbdElWahab, M. & Kamal, K. Role of some biological pollutants in relation to disease occurrence in *Oreochromis Niloticus*. *Suez Canal Vet. Med. J.*** 22**, 247–257 (2017).

[83] Ghosh, D. et al. Characterization of a hemolytic and antibiotic-resistant *Pseudomonas aeruginosa* strain S3 pathogenic to fish isolated from Mahananda River in India. *PLoS One*** 28**, 19. 10.1371/journal.pone.0300134 (2024). 10.1371/journal.pone.0300134 PMC10977779 38547304

[84] Tartor, Y. H. et al. Virulotyping and genetic diversity of *Aeromonas hydrophila* isolated from Nile tilapia (Oreochromis niloticus) in aquaculture farms in Egypt. *Aquaculture*** 541**, 736781. 10.1016/j.aquaculture.2021.736781 (2021).

[85] Abou Elez, R. M. M. et al. Antimicrobial resistance of *Salmonella enteritidis* and *Salmonella typhimurium* isolated from laying hens, table eggs, and humans with respect to antimicrobial activity of biosynthesized silver nanoparticles. *Animals (Basel) ***11**, 3554. 10.3390/ani11123554 (2021). 10.3390/ani11123554 PMC8698057 34944331

[86] Cepas, V. & Soto, S. M. Relationship between virulence and resistance among Gram-negative Bacteria. *Antibiotics*** 9**, 719 (2020). 33092201 10.3390/antibiotics9100719 PMC7589547

