# Supplementary material for: Resistance patterns, virulence determinants, and biofilm genes of multidrug-resistant Pseudomonas aeruginosa isolated from fish and fish handlers
Source: Sci Rep. 2024 Oct 14;14:24063. doi: 10.1038/s41598-024-73917-4 (PMC11473961; doi:10.1038/s41598-024-73917-4)
Supplement: Supplementary file 1 — Supplementary Material 1 [file 41598_2024_73917_MOESM1_ESM.docx]

**Resistance patterns, virulence determinants, and biofilm genes of multidrug-resistant *Pseudomonas aeruginosa* isolated from fish and fish handlers**

**Rasha M. M. Abou Elez^1^*, Eman Mohamed Fayek Zahra^2^, Rasha M. A. Gharieb^1^, Mohamed Elsayed Mohamed Mohamed^1^, Mohamed Samir^1^, Alaaeldin Mohamed Saad^1^, Abdallah Mohamed Amin Merwad^1^**

^1^ Department of Zoonoses, Faculty of Veterinary Medicine, Zagazig University, Egypt.

*** Correspondence:**Rasha M. M. Abou Elez ([rmmohamed@zu.edu.eg](mailto:rmmohamed@zu.edu.eg))

**Table S 1.** Primers' sequences and PCR cycling conditions used in this study.

| **Target gene** | **Primers' sequences**  **5^\^-3^\^** | **Amplified product (bp)** | **Primary**  **denaturation** | **Amplification (35 cycles)** | | | **Final extension** | **Reference** |
| --- | --- | --- | --- | --- | --- | --- | --- | --- |
|  |  |  |  | **Secondary denaturation** | **Annealing** | **Extension** |  |  |
| 16S r*DNA* | GGGGGATCTTCGGACCTCA | 956 | 94˚C  5 min. | 94˚C  30 sec. | 52˚C  40 sec. | 72˚C  50 sec. | 72˚C  10 min. | Spilker et al. **^27^** |
|  | TCCTTAGAGTGCCCACCCG |  |  |  |  |  |  |  |
| *las*B | ACAGGTAGAACGCACGGTTG | 1220 | 94˚C  5 min. | 94˚C  30 sec. | 54˚C  40 sec. | 72˚C  1min. | 72˚C  12 min. | Finnan et al. **^20^** |
|  | GATCGACGTGTCCAAACTCC |  |  |  |  |  |  |  |
| *tox*A | GACAACGCCCTCAGCATCACCAGC | 396 | 94˚C  5 min. | 94˚C  30 sec. | 55˚C  40 sec. | 72˚C  40 sec. | 72˚C  10 min. | Matar et al. **^33^** |
|  | CGCTGGCCCATTCGCTCCAGCGCT |  |  |  |  |  |  |  |
| *exo*U | CCGTTGTGGTGCCGTTGAAG | 134 | 94˚C  5 min. | 94˚C  30 sec. | 55˚C  30 sec. | 72˚C  30 sec. | 72˚C  12 min. | Winstanley et al. **^34^** |
|  | CCAGATGTTCACCGACTCGC |  |  |  |  |  |  |  |
| *opr*L | ATGGAAATGCTGAAATTCGGC | 504 | 94˚C  5 min. | 94˚C  30 sec. | 55˚C  40 sec. | 72˚C  40 sec. | 72˚C  10 min. | Xu et al. **^35^** |
|  | CTTCTTCAGCTCGACGCGACG |  |  |  |  |  |  |  |
| *psl*A | TCCCTACCTCAGCAGCAAGC | 656 | 94˚C  5 min. | 94˚C  30 sec. | 60˚C  40 sec. | 72˚C  45 sec. | 72˚C  10 min. | Ghadaksaz et al. **^36^** |
|  | TGTTGTAGCCGTAGCGTTTCTG |  |  |  |  |  |  |  |
| *pel*A | CATACCTTCAGCCATCCGTTCTTC | 786 | 94˚C  5 min. | 94˚C  30 sec. | 60˚C  40 sec. | 72˚C  45 sec. | 72˚C  10 min. |  |
|  | TCCCTACCTCAGCAGCAAGC |  |  |  |  |  |  |  |
| *las*R | CTGTGGATGCTCAAGGACTAC | 133 | 94˚C  5 min. | 94˚C  30 sec. | 55˚C  30 sec. | 72˚C  30 sec. | 72˚C  7 min. | Saleh et al. **^37^** |
|  | AACTGGTCTTGCCGATGG |  |  |  |  |  |  |  |
| *las*I | ATGATCGTACAAATTGGTCGGC | 606 | 94˚C  5 min. | 94˚C  30 sec. | 56˚C  40 sec. | 72˚C  45 sec. | 72˚C  10 min. | Bratu et al. **^38^** |
|  | GTCATGAAACCGCCAGTCG |  |  |  |  |  |  |  |
| *rhl*R | GCCAGCGTCTTGTTCGG | 160 | 94˚C  5 min. | 94˚C  30 sec. | 60˚C  30 sec. | 72˚C  30 sec. | 72˚C  7 min. | Saleh et al. **^37^** |
|  | CGGTCTGCCTGAGCCATC |  |  |  |  |  |  |  |

**Table S 2.** Prevalence and distribution of *Pseudomonas* spp. isolates (%) in fish and fish handlers.

| **Host** | **Examined number** | **liver** | **Muscle** | **total** |
| --- | --- | --- | --- | --- |
| Nile tilapia | 53 | 17 (32.1) | 15 (28.3) | 32 (60.4) |
| Golden grey mullet | 52 | 19 (36.5) | 15 (28.8) | 34 (65.4) |
| **Total** | **105** | **36 (34.3)** | **30 (2.9)** | **66 (62.9)** |
| Mediterranean horse mackerel | 50 | 17 (34) | 12 (24) | 29 (58) |
| Striped red mullet | 71 | 32 (45.1) | 10 (14.1) | 42 (59.2) |
| **Total** | **121** | **49 (40.5)** | **22 (18.2)** | **71 (58.7)** |
| **Grand Total** | **226** | **85 (37.6)** | **52 (23)** | **137 (60.6)** |
| Human | 50 |  |  | 23 (46) |


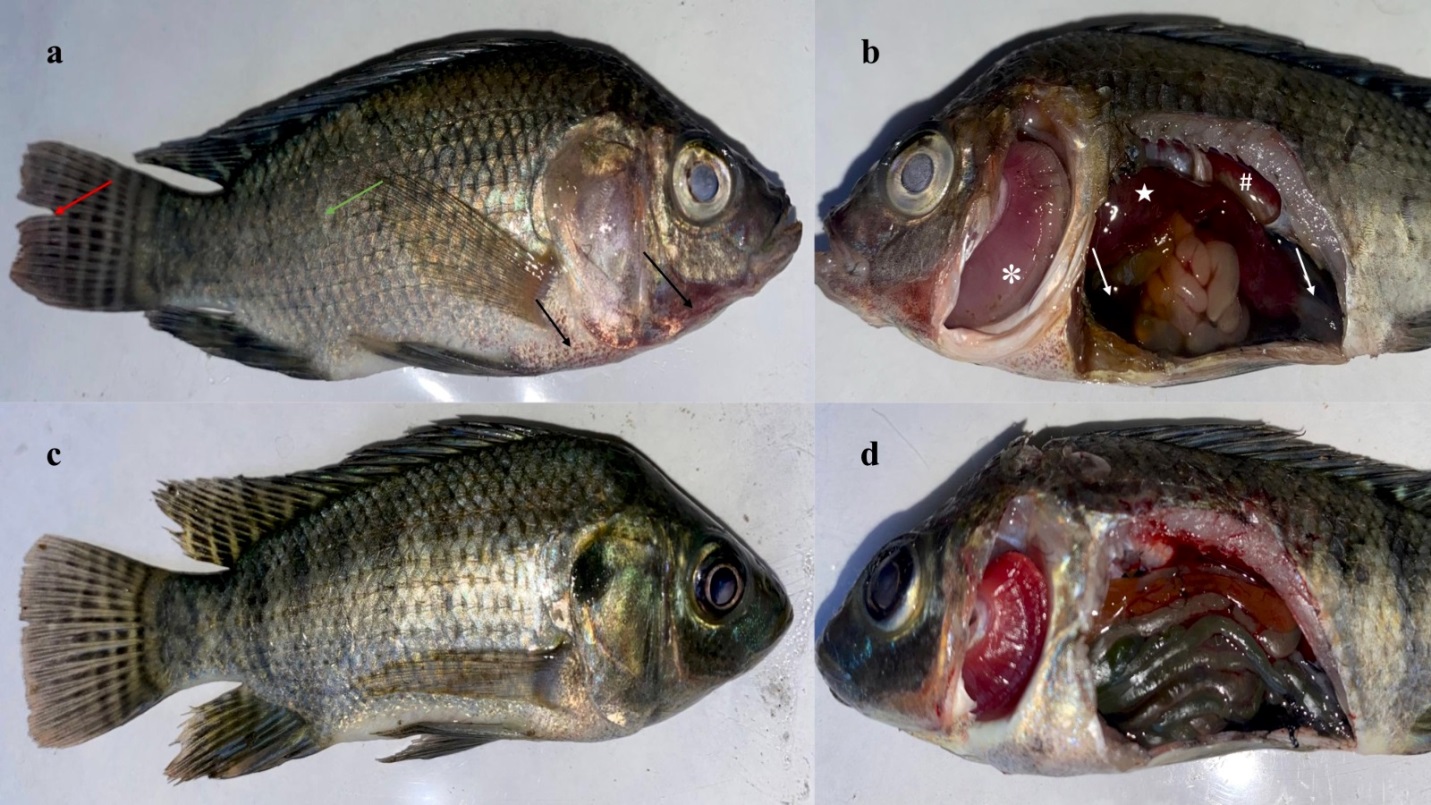


**Figure S2.** Clinical and post-mortem examination of artificially infected fish (**a**) Nile tilapia showing scattered spots of hemorrhages, especially at the ventral part of the head and abdomen (black arrows), fins erosion (red arrow), and detached scales (green arrow), (b) on external body surfaces, especially at the ventral part of the abdomen and around the vent (white arrows), (**b**) Nile tilapia showing necrotic gills (^✽^), serous fluid exudate (white arrows), friable liver (^★^), and congested kidney (^#)^), (c) and (d) Control apparent healthy Nile tilapia .
